# Rapid, low-cost, and portable molecular detection of HIV-1 subtype B and HCV 1a directly from blood

**DOI:** 10.1128/spectrum.00416-26

**Published:** 2026-08-05

**Authors:** David M. Tang, Karen Michalak, Kelvin Nguyen, Philip Lacap, John Kim, Paul J. McLaren

**Affiliations:** 1 Sexually Transmitted and Bloodborne Infections Division, National Microbiology Laboratories, JC Wilt Infectious Diseases Research Centre, Public Health Agency of Canada 384849, Winnipeg, Manitoba, Canada; 2 Department of Medical Microbiology and Infectious Diseases, University of Manitoba 8664, Winnipeg, Canada; Indiana University School of Medicine, Indianapolis, Indiana, USA

**Keywords:** HIV, HCV, RT-LAMP, isothermal amplification, molecular test direct from blood, CRISPR diagnostics, lyophilized master mix, decentralized testing

## Abstract

Accessible molecular diagnostics for human immunodeficiency virus-1 (HIV-1) and hepatitis C virus (HCV) remain constrained by the cost and infrastructure requirements of existing molecular point-of-care platforms. In this study, we describe a portable, proof-of-concept molecular workflow with a total runtime of 32 min for the simultaneous qualitative detection of HIV-1 subtype B and HCV genotype 1a directly from contrived whole blood. The workflow employs a battery-powered heating device, ambient-stable reagents, and lateral-flow dipstick readout. Contrived whole blood samples were diluted twofold with nuclease-free water and added directly to rehydrated reverse-transcription loop-mediated amplification (RT-LAMP) reactions without nucleic acid extraction, and reaction parameters were systematically varied to determine lower limits of detection. Both viral targets were consistently detected at clinically relevant input levels following a 30 min isothermal amplification step, with visual lateral flow results available within 2 min. In addition, CRISPR-Cas12a detection was used as an orthogonal method to verify RT-LAMP target amplicons during assay development. Together, this work establishes the analytical feasibility of a rapid, low-cost, portable molecular workflow and provides a foundation for future development of decentralized HIV-1 and HCV molecular diagnostics.

**IMPORTANCE:**

Ongoing changes in human immunodeficiency virus (HIV) and hepatitis C virus (HCV) epidemiology highlight the need for more adaptable and accessible diagnostic tools. In this study, we developed a fast, low-cost test that detects HIV-1 and HCV from a small blood sample. The test is designed to work outside traditional laboratories, using minimal equipment. It does not require complex sample preparation, refrigeration, or expensive machines, and produces results in about 30 min. The workflow combines a rapid genetic amplification method with an easy-to-read test strip and a small, battery-powered heater, making it portable and suitable for use in low-resource or remote settings. An additional molecular method was used during development to confirm that the test was accurately detecting the correct viral targets. By focusing on simplicity, speed, and affordability, this work aims to expand access to reliable HIV and HCV testing in settings where these infections often occur together and laboratory access is limited.

## INTRODUCTION

A substantial number of people living with human immunodeficiency virus-1 (HIV-1) or hepatitis C virus (HCV) remain undiagnosed, undermining global elimination efforts. Recent estimates indicate that 79% of people with HCV and 13% of people with HIV-1 are unaware of their infection (1, 2). Diagnostic gaps are even more pronounced in populations with elevated transmission risk, such as infants born to mothers living with HIV-1 and people who inject drugs (PWID). In 2024, approximately 37% of children living with HIV-1 had not received a diagnosis (3). Among PWID, nearly 90% experienced missed opportunities for HIV-1 or HCV testing (4). Such gaps are concerning given that outbreak investigations consistently report high HCV/HIV-1 coinfection prevalence among PWID, often exceeding 70% (5). Failure to diagnose infections during the early stages, when viral loads peak and transmission risk is highest, further exacerbates the spread of both viruses (6). Individuals who remain undiagnosed and untreated also face substantially elevated risks of morbidity and mortality (7, 8). Recognizing these challenges, the Joint United Nations Programme on HIV/AIDS (UNAIDS) and the World Health Organization have identified expanded and accessible testing as a foundational requirement for achieving the 2030 HIV-1 and HCV elimination targets (2, 9).

Molecular testing is essential for the accurate diagnosis of HIV-1 and HCV, providing substantially greater sensitivity and earlier detection than serological methods. This is particularly important in newborn screening, where maternally transferred antibodies can persist for months and render serology unreliable (10). Molecular assays are also required to confirm active HCV infection, as a positive antibody test may reflect past exposure rather than ongoing infection (11). In addition, breakthrough infections among individuals receiving long-acting cabotegravir for pre-exposure prophylaxis (PrEP) have demonstrated that HIV-1 viral load may remain low or undetectable for extended periods before rebounding to detectable levels, underscoring the need for frequent, repeated molecular testing (12). However, such intensive testing strategies are currently constrained by the high cost and limited accessibility of conventional molecular platforms (13–15). Together, these challenges highlight an urgent need for affordable, accessible molecular diagnostics that can support repeated testing and timely detection of infection, particularly in resource-limited settings and among populations requiring ongoing monitoring.

Fully automated molecular point-of-care (MPOC) cartridge systems that integrate nucleic acid extraction, amplification, and detection have substantially advanced efforts to identify previously undiagnosed HIV-1 and HCV infections, particularly within key populations, including gay, bisexual, trans, and other men who have sex with men; indigenous communities; people of African, Caribbean, and Black ethnicities; sex workers; and people who inject drugs (16). However, despite the operational simplicity and minimal hands-on requirements of these MPOC systems, widespread adoption in resource-limited settings remains hindered by the high capital cost of MPOC systems and the recurring expense of single-use cartridges (15). Even with negotiated buy-down agreements, many low- and middle-income countries continue to face substantial financial barriers to expanding access (17). Further hindrances include prolonged assay run times (typically 60–90 min) and restricted portability due to instrument size, weight, and dependence on continuous plug-in power (15).

Reverse-transcription loop-mediated amplification (RT-LAMP) is a promising approach for expanding MPOC testing access for HIV-1 and HCV (18–28). This one-step nucleic acid amplification method operates at a constant temperature and therefore requires only simple heating devices, such as a heating block (29). RT-LAMP can amplify viral nucleic acids directly from crude samples within 30 min and supports low-cost analog readouts, including lateral-flow dipsticks, removing the need for expensive fluorescence instrumentation (18, 29–31). Collectively, these features substantially reduce hardware complexity, sample-preparation requirements, and overall run time. Although RT-LAMP has been applied to HIV-1 and HCV detection, its use in MPOC tests remains limited. A key barrier has been the need for cold-chain storage of reagents (18, 19, 21, 22, 24, 27, 28, 32, 33). Recent advances, however, have introduced off-the-shelf RT-LAMP master mixes that co-lyophilize all essential reaction components (except the primer set), thereby enhancing field deployability.

In this study, we sought to develop and analytically evaluate a 32 min total-runtime, portable, low-cost, proof-of-concept molecular diagnostic workflow for the simultaneous qualitative detection of HCV genotype 1a and HIV-1 subtype B directly from contrived whole blood. The workflow operates without cold-chain storage and combines extraction-free RT-LAMP, lateral-flow dipstick readout, and a handheld battery-powered heating device. In addition, we evaluated CRISPR-Cas12a detection as an orthogonal approach to verify RT-LAMP amplicon specificity during assay development.

## RESULTS

### Screening primer sets and verifying target amplicons

Three HCV (21, 22, 27) and four HIV-1 primer sets (20, 25, 26, 28) (Table S1) were screened using an off-the-shelf lyophilized master mix (LyoPrime WarmStart Fluorescent LAMP/RT-LAMP Mix [with UDG]) and serially diluted extracted viral RNA. Each primer set was evaluated based on its time-to-positive (TTP) and lower limit of detection (LLOD). Screening was performed under a single reaction condition, consisting of one incubation temperature (62°C) and one betaine concentration (0.5 M). Betaine was included because prior evidence indicated that it enhances detection sensitivity and delays the onset of non-target amplicons in cold-stored custom master mixes (Table S2) (34). Among the primer sets tested, HCV-K12 (21) and HIV-1-Z14 (28) exhibited the lowest LLODs and the fastest average TTPs (within 30 min) (Table S3). Given the tendency of RT-LAMP to generate non-target amplicons depending on the master mix formulation (Fig. S1 and Tables S2 and S4) (34, 35), we incorporated CRISPR-Cas12 fluorescence detection with custom-designed guide RNAs (Tables S5 and S6) as a rapid and cost-effective post-amplification method for confirming the presence of target amplicons (36). This approach revealed that when using the off-the-shelf lyophilized master mix, non-target amplicons occurred very infrequently and exhibited delayed TTPs (≥55 min) compared to target amplicons (Tables S7 and S8).

### Performance of extraction-free RT-LAMP in whole blood

Challenges in molecular diagnostics for bloodborne pathogens include the cost, hands-on time, and hardware requirements associated with nucleic acid extraction from blood. To address these challenges, we built on prior studies that employed extraction-free RT-LAMP, in which diluted pathogenic blood samples are added directly to the reaction (18, 24, 29). Here, we assessed the feasibility of developing an extraction-free HCV RT-LAMP assay using an off-the-shelf lyophilized master mix, as its compatibility with blood samples containing viral pathogens (HIV-1 and HCV) had not been established. We prepared a contrived whole blood sample containing patient-derived plasma with HCV genotype 1a (5.47 log_10_ IU/mL) to evaluate the effects of different blood diluents and dilution ratios on extraction-free HCV RT-LAMP performance. Nuclease-free water yielded the fastest average TTP (16.2 ± 0.4 min) at a 1:1 dilution ratio (Table 1). Notably, adding 5 µL of twofold-diluted blood, rather than 5 µL of undiluted blood, to a 20 µL extraction-free HCV RT-LAMP reaction resulted in more robust and consistent amplification.

**TABLE 1:** Effect of blood diluents and dilution ratios on extraction-free HCV RT-LAMP performance using a contrived whole blood sample positive for HCV genotype 1a (5.47 log_10_ IU/mL)^*d*^

Blood diluent	Dilution ratio^*a*^	Blood concentration in reaction (% vol/vol)^*b*^	Detectable TTP (replicates)	Mean TTP (min) ±SD	Positive CRISPR detection^*c*^
None	N/A	20	1/3	25.5	1/3
Nuclease-free water	1:1	10	3/3	16.2 ± 0.4	3/3
	1:3	5	3/3	21.7 ± 1.4	3/3
RBC lysing buffer	1:1	10	3/3	17.1 ± 2.4	3/3
	1:3	5	3/3	21.2 ± 3.5	3/3
ACK lysing buffer	1:1	10	3/3	20.8 ± 6.4	3/3
	1:3	5	3/3	27.1 ± 5.5	3/3

^*a*^ Parts of contrived HCV genotype 1a-positive whole blood (5.47 log_10_ IU/mL) to parts of diluent.

^*b*^ 5 µL of undiluted or diluted contrived whole blood added into a 20 µL extraction-free HCV RT-LAMP reaction.

^*c*^ CRISPR-Cas12a fluorescence detection performed on 2 µL of post-amplification material to confirm the presence of target amplicons.

^*d*^ Abbreviations: RBC, red blood cell; ACK, ammonium-chloride-potassium; TTP, time-to-positive; min, minutes; SD, standard deviation; N/A, not applicable.

### Lower limit of detection for extraction-free HCV and HIV-1 RT-LAMP with lateral-flow detection

**Fig 1:**
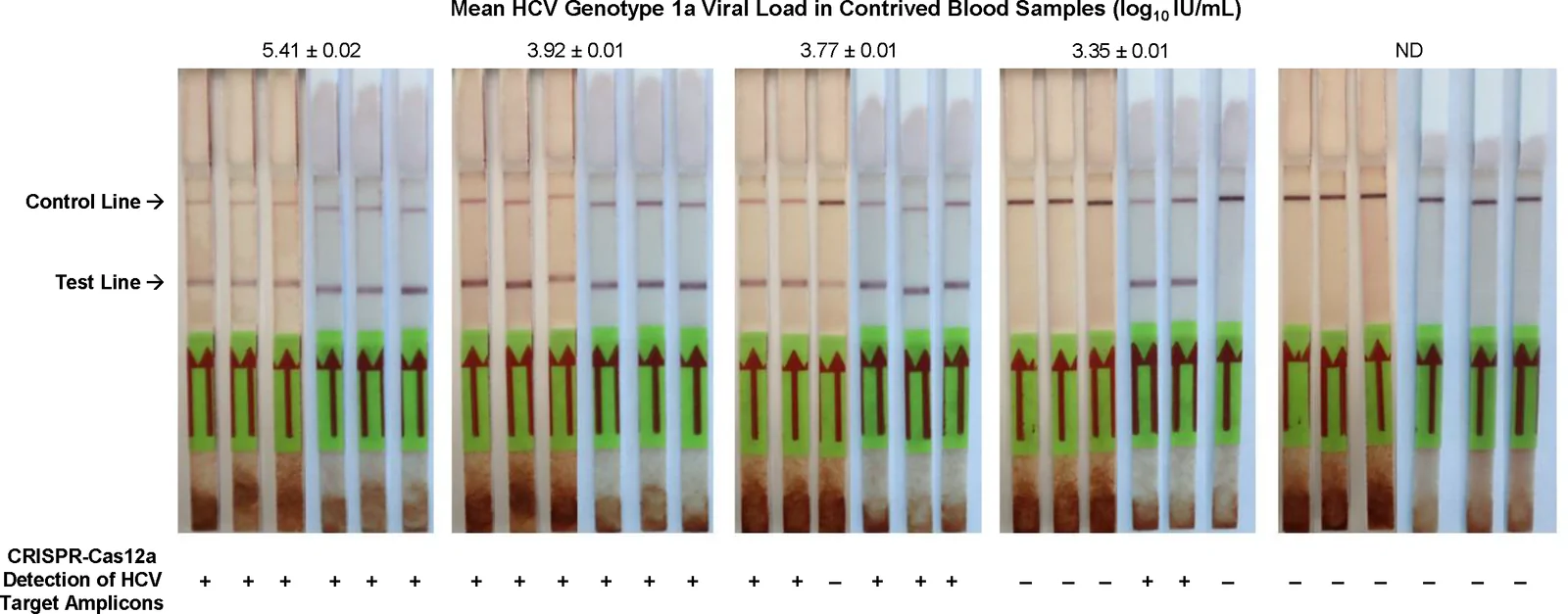
Lower limit of detection of HCV genotype 1a using an optimized extraction-free HCV RT-LAMP assay with lateral-flow readout. Contrived whole blood samples were prepared using patient-derived HCV genotype 1a plasma reference material, and viral loads were quantified via the Xpert HCV VL Fingerstick assay. Samples were diluted twofold in nuclease-free water, with a 5 µL aliquot of the diluted mixture added directly into a 20 µL HCV RT-LAMP reaction. Following a 30 min incubation at 62°C in the presence of 0.5 M betaine, target amplification was evaluated using two readout methods: CRISPR-Cas12a fluorescence detection and lateral-flow readout. Biotin- and 6-carboxyfluorescein-labeled RT-LAMP amplicons were visualized at the test line within 2 min, with the control line confirming proper dipstick function. ND (not detected) indicates the contrived whole-blood negative control, verified as negative via the Xpert HCV VL Fingerstick assay.

Based on the optimal RT-LAMP conditions identified using serially diluted extracted viral RNA (Tables S7 and S8), we evaluated whether these conditions remained optimal when applied to twofold-diluted contrived whole blood containing HCV genotype 1a or HIV-1 subtype B. For the HCV-K12M primer set, the optimal condition remained 62°C with 0.5 M betaine, yielding an assay LLOD of 3.77 log_10_ IU/mL (Table 2). At this concentration, only two of six replicates generated a detectable TTP via real-time fluorescence; however, lateral-flow dipsticks successfully detected biotin- and 6-carboxyfluorescein-labeled amplicons across all six replicates (Fig. 1). Notably, one replicate produced a faint test band on the lateral-flow dipstick but was negative by CRISPR-Cas12a fluorescence detection. We attributed this discrepancy to low amplicon yield. Given the limited input volume (2 µL) of post-RT-LAMP product introduced into the 98 µL CRISPR-Cas12a reaction mixture, the target amplicon concentration was likely insufficient to generate a detectable fluorescence signal.

**TABLE 2:** Lower limit of detection for the extraction-free HCV RT-LAMP assay under various reaction conditions^*c*^

RT-LAMP reaction conditions		LLOD for HCV genotype 1a in contrived blood (log_10_ IU/mL)^*a*^	Replicates detected		
30 min incubation temp. (°C)	Betaine conc. (M)		Detectable TTP	2 min lateral flow	CRISPR detection^*b*^
62	0	3.92	1/3	3/3	3/3
	0.5	3.77	2/6	6/6	5/6
63	0	5.41	2/3	3/3	3/3
	0.5	3.92	0/3	3/3	3/3

^*a*^ The viral loads of contrived whole blood samples were 5.41, 3.92, 3.77, 3.35, and 3.09 log_10_ IU/mL, as quantified using the Xpert HCV VL Fingerstick test (60 min runtime), whereas the HCV-negative sample was below the limit of detection. The LLOD experiment performed at 62**°**C in the presence of 0.5 M betaine was repeated on a separate day. The reported LLOD represents the mean of two independent viral load measurements (SD ± 0.01 log_10_ IU/mL).

^*b*^ CRISPR-Cas12a fluorescence detection performed on 2 µL of post-amplification material to confirm the presence of target amplicons.

^*c*^ Abbreviations: Temp., temperature; Conc., concentration; TTP, time-to-positive; LLOD, lower limit of detection.

**Fig 2:**
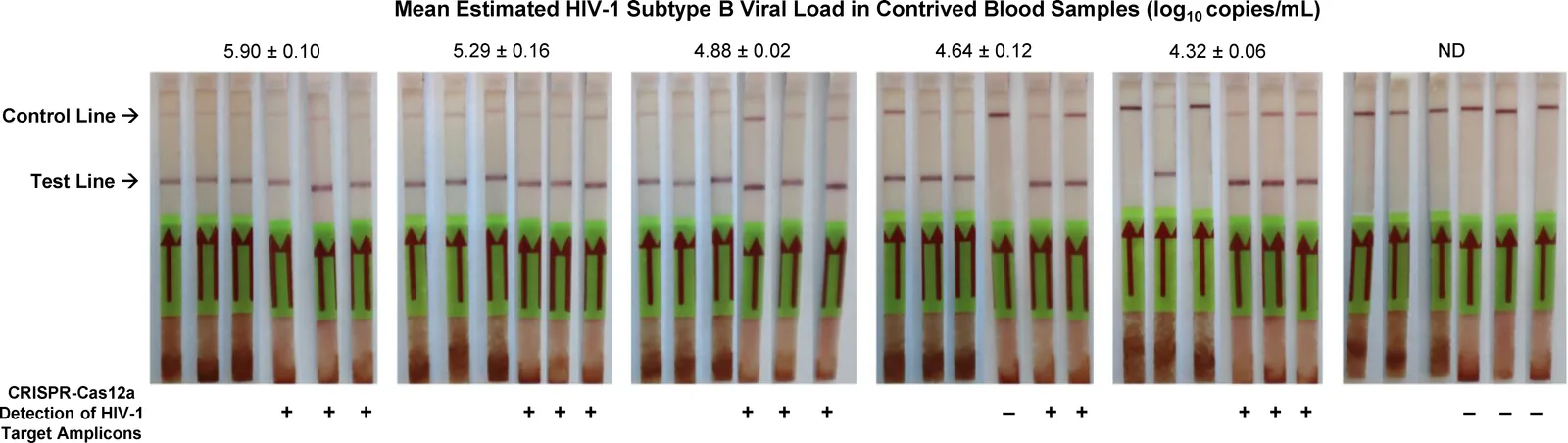
Lower limit of detection of HIV-1 subtype B using an optimized extraction-free HIV-1 RT-LAMP assay with lateral-flow readout. Contrived whole blood samples were prepared using HIV-1 subtype B plasma reference material, with viral loads estimated via the Xpert HIV-1 Qual assay. Samples were diluted twofold using nuclease-free water, with a 5 µL aliquot of the diluted mixture added directly into a 20 µL HIV-1 RT-LAMP reaction. Following a 30 min incubation at 61°C in the presence of 0.5 M betaine, target amplification was assessed using two readout methods described in Fig. 1. ND (not detected) indicates the contrived whole-blood negative control, verified as negative via the Xpert HIV-1 Qual assay.

For the HIV-1-Z14M primer set, the optimal condition identified using extracted RNA (61°C and 0 M betaine) did not yield the lowest LLOD when applied to the contrived whole blood samples. Instead, incubation at 61°C with 0.5 M betaine produced the lowest LLOD, corresponding to an estimated concentration of 4.88 log_10_ copies/mL (Table 3). At this viral load, none of the six replicates generated a detectable TTP via real-time fluorescence; however, lateral-flow dipsticks successfully detected target amplicons across all replicates (Fig. 2). While independent experimental runs yielded completely consistent results for the primary lateral-flow readout, an inadvertent discrepancy in the downstream fluorescence reporter molecule restricted the corresponding confirmatory CRISPR-Cas12a validation data to a single representative data set. We next assessed whether the optimized extraction-free HIV-1 RT-LAMP assay could detect integrated proviral DNA, an important biomarker of HIV infection, particularly in contexts where serological testing is unreliable or plasma viral RNA is undetectable (37, 38). Genomic DNA from 8E5 cells was extracted, serially diluted, and 5 µL was added directly to the optimized reaction. Under these conditions, the LLOD was 0.078 ng with a mean TTP 23.1 ± 3.8 min (Table S9), corresponding to approximately 12 HIV-1 proviral DNA copies based on a single integrated copy per individual 8E5 cell (39, 40).

**TABLE 3:** Lower limit of detection for the extraction-free HIV-1 RT-LAMP assay under various reaction conditions^*c*^

RT-LAMP reaction conditions		Mean estimated HIV-1 subtype B viral load at the LLOD in contrived blood ±SD (log_10_ copies/mL)^*a*^	Replicates detected		
30 min incubation temp. (°C)	Betaine conc. (M)		Detectable TTP	2 min lateral flow	CRISPR detection^*b*^
61	0	5.29 ± 0.16	0/6	6/6	3/3
	0.5	4.88 ± 0.02	0/6	6/6	3/3
62	0	5.90 ± 0.10	2/6	6/6	3/3
	0.5	5.29 ± 0.16	0/6	6/6	3/3

^*a*^ The mean estimated viral loads of the contrived whole blood samples were 5.90, 5.29, 4.88, 4.64, and 4.32 log_10_ copies/mL, whereas the HIV-1-negative sample was below the limit of detection. Viral loads were estimated by back-calculating from the cycle-threshold values of the Xpert HIV-1 Qual (93 min runtime) via the linear regression equation provided in the manufacturer’s instructions for use. Experiments were repeated on a separate day.

^*b*^ CRISPR-Cas12a fluorescence detection performed on 2 µL of post-amplification material to confirm the presence of target amplicons.

^*c*^ Abbreviations: Temp., temperature; Conc., concentration; SD, standard deviation; TTP, time-to-positive; LLOD, lower limit of detection.

### Portable diagnostics

**Fig 3:**
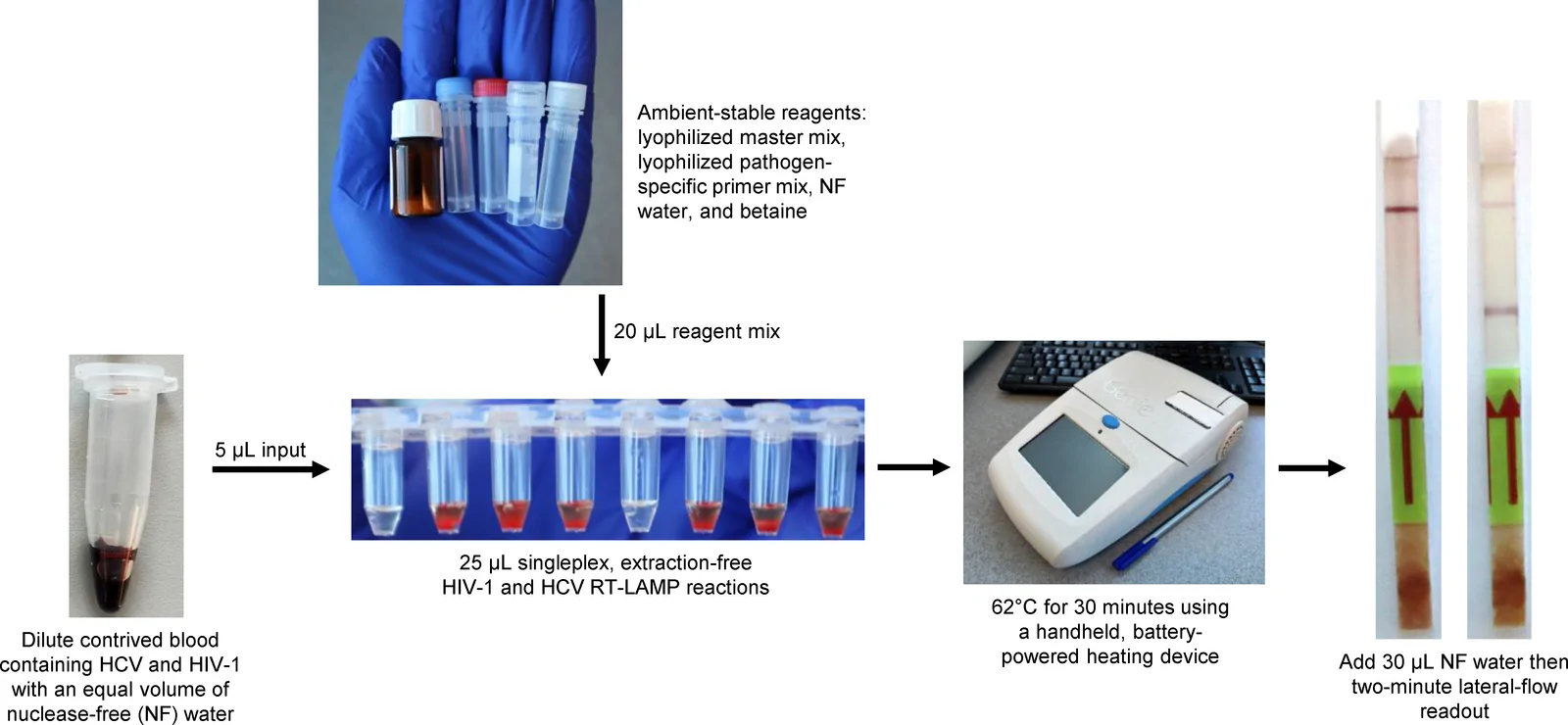
Schematic of a portable 32 min dual-pathogen diagnostic workflow. The workflow enables simultaneous detection of HIV-1 (subtype B) and HCV (genotype 1a) directly from contrived whole blood samples using ambient-stable reagents, a handheld battery-powered heating device, and lateral-flow readout.

**Fig 4:**
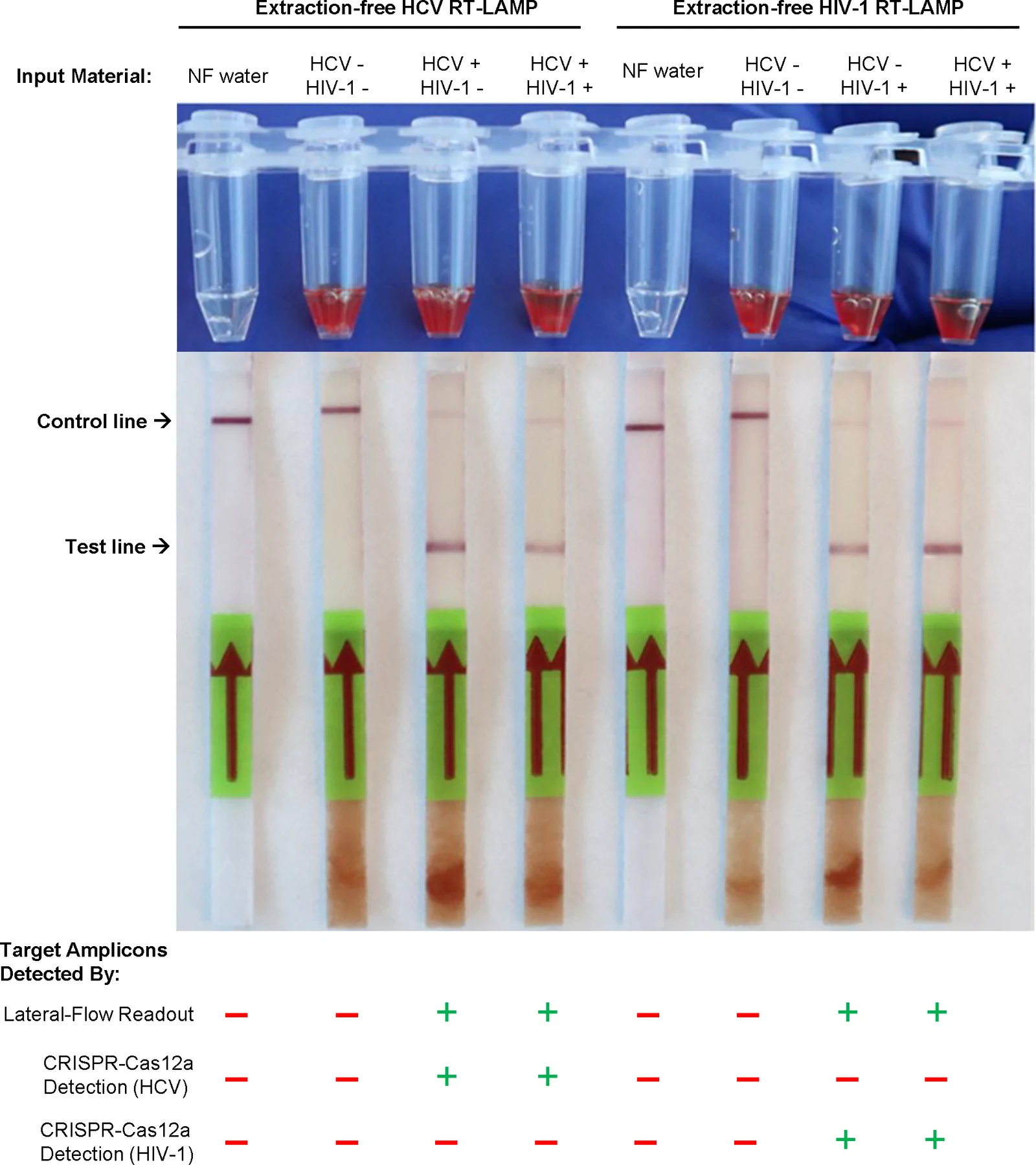
Thirty-two-minute workflow for the simultaneous evaluation of four contrived whole blood samples using ambient-stable reagents, a handheld battery-powered heating device, and lateral-flow readout**.** The following contrived samples were evaluated: (i) negative for both HCV genotype 1a and HIV-1 subtype B; (ii) HCV genotype 1a (5.05 log_10_ IU/mL); (iii) HCV genotype 1a (5.23 log_10_ IU/mL) and HIV-1 subtype B (5.46 log_10_ copies/mL); and (iv) HIV-1 subtype B (5.46 log_10_ copies/mL). HCV viral loads were quantified using the Xpert HCV VL Fingerstick assay, and the HIV-1 viral load was estimated using the Xpert HIV-1 Qual assay. An aliquot of each sample was diluted twofold with nuclease-free (NF) water, and 5 µL of the diluted mixture was directly added into singleplex, extraction-free HCV and HIV-1 RT-LAMP reactions. Following a 30 min incubation at 62°C using a battery-powered heating device, target amplicons were detected via two readout methods described in Fig. 1.

To demonstrate proof-of-concept field deployability, ambient-stable reagents were utilized throughout, and reactions were incubated in a handheld, battery-powered heating device (Fig. 3). A single eight-tube strip was used to simultaneously incubate singleplex, extraction-free HCV and HIV-1 RT-LAMP reactions supplemented with a final concentration of 0.5 M betaine at 62°C for 30 min. Coupled with a 2 min lateral-flow dipstick readout, the total assay runtime was 32 min to evaluate four distinct contrived whole blood samples, including one sample containing both HIV-1 subtype B and HCV genotype 1a (Fig. 4). Assay specificity was further assessed by demonstrating that the extraction-free HCV RT-LAMP assay did not generate target amplicons from HIV-1 subtype B-containing samples, and conversely, the extraction-free HIV-1 RT-LAMP assay did not generate target amplicons from HCV genotype 1a-containing samples (Fig. S2).

## DISCUSSION

Here, we describe a rapid, portable proof-of-concept molecular workflow enabling simultaneous detection of HCV and HIV-1 directly from contrived whole blood samples with visual readout. The end-to-end assay requires a total runtime of 32 min and relies on ambient-stable reagents, a battery-powered heating device, and lateral-flow dipsticks. While prior studies have demonstrated the feasibility of extraction-free RT-LAMP for HIV-1 or HCV detection using direct blood input, many of these approaches remain limited by a reliance on cold-chain infrastructure for reagent stability and plug-in power, as well as lengthy incubation times and complex amplicon visualization strategies (18–20, 22, 24, 26–28, 32, 33). These constraints have limited their practical suitability for near-patient or point-of-care use, particularly in resource-limited settings (18, 24).

Prior to this study, it was unknown whether RT-LAMP reactions using the selected off-the-shelf lyophilized master mix could tolerate pathogen-containing whole blood as a direct input material. Initial experiments revealed that extraction-free HCV RT-LAMP was strongly inhibited at a final concentration of 20% vol/vol whole blood contrived with HCV genotype 1a, likely due to the high levels of hemoglobin and hematin, which are well-known inhibitors of DNA polymerase activity (41). Consequently, we systematically evaluated alternative blood diluents and dilution ratios to optimize amplification performance. Diluting contrived whole blood samples with an equal volume of nuclease-free water, followed by the direct input of 5 µL into the RT-LAMP reaction (final blood concentration of 10% vol/vol), yielded the fastest average TTP while maintaining robust amplification across all technical replicates. We propose that nuclease-free water confers two key advantages: first, the absence of chemical components that could interfere with amplification; and second, the creation of a hypotonic environment that promotes the osmotic lysis of red blood cells (RBCs), thereby disrupting the dense sample matrix and reducing steric hindrance to improve overall nucleic acid accessibility (24, 42). Furthermore, within this extraction-free workflow, the incubation temperature inherent to RT-LAMP likely further facilitates virion disruption and viral RNA release (43–45).

To simplify target amplicon detection, we evaluated lateral-flow dipsticks following extraction-free HCV and HIV-1 RT-LAMP reactions. This approach offers several advantages for near-patient or point-of-care utility. Primarily, lateral-flow dipsticks eliminate reliance on dedicated hardware or software for assay readout and data analysis, thereby enhancing diagnostic autonomy, affordability, and portability. Furthermore, the visual readout is rapid (2 min) and mitigates the false-negative results frequently observed with real-time fluorescence in this study. At blood concentrations exceeding 10% vol/vol, high levels of hemoglobin and hematin (known PCR and LAMP inhibitors) likely quenched the fluorescent signal or physically obstructed optical detection due to elevated sample turbidity, leading to undetectable TTP values. While lateral-flow dipsticks enabled rapid, equipment-free detection, their use during high-throughput assay development was cost-prohibitive due to the large number of biological and technical replicates required. Accordingly, we utilized CRISPR-Cas12a detection with custom-designed guide RNAs as a scalable, cost-efficient orthogonal method to verify RT-LAMP amplicons before transitioning to the final lateral-flow format.

This study establishes clinically relevant LLODs for HCV genotype 1a and HIV-1 subtype B using a simplified molecular diagnostic workflow that proceeds directly from contrived whole blood to visual lateral-flow dipstick readout, requiring ambient-stable reagents and a total runtime of 32 min.

Among the HCV genotypes evaluated, genotype 1a exhibited the most sensitive LLOD at 3.77 log_10_ IU/mL, indicating that the assay is capable of detecting the majority of clinical HCV genotype 1a infections. Supporting this, a large-scale study involving over 66,000 individuals with chronic HCV infection across 12 countries reported that 95% had viral loads ≥ 3.52 log_10_ IU/mL (46). While genotype 1b was detected with comparable sensitivity, reduced or absent amplification was observed for genotypes 2b and 3a (Fig. S3 and Table S10). *Post hoc* sequence alignment revealed several primer-template mismatches within these specific genotypes (Tables S11), elucidating the underlying cause of the reduced amplification efficiency. Although the HCV-K12 primer set was originally described as pan-genotypic (21), our findings indicate that sequence-specific optimization may be necessary to achieve high sensitivity within this rapid, extraction-free, and lyophilized RT-LAMP workflow. These findings establish an analytical benchmark for the development of primer sets capable of achieving comparable or improved sensitivity (3.77 log_10_ IU/mL) across the most clinically relevant genotypes while remaining compatible with this simplified workflow. While further validation is required to evaluate the workflow as a first-line molecular screening tool in low-prevalence populations, its immediate utility lies in high-prevalence settings, where viral loads frequently exceed 6 log_10_ IU/mL (47).

For HIV-1 subtype B detection from contrived whole blood, the LLOD was estimated to be 4.88 log_10_ copies/mL. Studies focused on viral dynamics during acute HIV-1 infection have shown that peak viremia frequently surpasses 5 log_10_ copies/mL, suggesting that this assay could serve effectively as a first-line screening tool in high-prevalence settings (48, 49). Similarly, studies have reported that the median viral load at birth is approximately 5.6 log_10_ copies/mL, increasing to ≥6 log_10_ copies/mL within the first months of life (50, 51). Given that approximately 52% of perinatally infected newborns die within the first year of life, early diagnosis and timely initiation of treatment are crucial, particularly in low-resource settings where access to testing may be limited and test turnaround times are prolonged (52).

To further evaluate the diagnostic utility of extraction-free HIV-1 RT-LAMP, we investigated whether the assay could detect integrated HIV-1 proviral DNA. Preliminary results indicated an estimated LLOD of 0.08 ng of genomic DNA from 8E5 cells, corresponding to approximately 12 copies of HIV-1 proviral DNA (39, 40). Additional studies are required to determine whether leukocytes are lysed through osmotic disruption following dilution with nuclease-free water or through heating during the RT-LAMP incubation step. If either mechanism effectively releases proviral DNA detectable by extraction-free HIV-1 RT-LAMP, diagnostic sensitivity could be further enhanced. This capability may increase the clinical utility of the assay for chronically HIV-1-infected individuals, those receiving long-acting PrEP, and HIV-1-infected children. Moreover, the low blood volume requirement of this workflow enables compatibility with minimally invasive sampling approaches, such as finger-prick or heel-prick collection, which is particularly advantageous for infant testing and use in resource-limited settings.

Despite therapeutic advances, HCV and HIV-1 infections continue to pose substantial global public health challenges, with co-infections disproportionately affecting marginalized and high-risk populations (53). Here, we demonstrate a proof-of-concept molecular workflow capable of simultaneously detecting HCV genotype 1a and HIV-1 subtype B directly from contrived whole blood within 32 min of runtime, using ambient-stable reagents, lateral-flow readout, and a commercially available handheld battery-powered heating device. To further improve accessibility, future work may focus on the development of a compact, palm-sized, non-proprietary heating device optimized for strip-tube formats. Based on current research-use pricing, the consumable cost per test is approximately $7 USD, including the lyophilized RT-LAMP reaction, primer set, betaine, and lateral-flow dipstick, and excluding capital equipment and control reactions. While lowering technical barriers and costs are essential for expanding testing access, the practical impact of this diagnostic workflow is best realized through its integration into a robust continuum of care that links results with immediate therapeutic intervention and longitudinal clinical management. Taken together, this streamlined diagnostic workflow provides a versatile platform that enables laboratories in endemic regions to scale up molecular testing capacity with increased operational autonomy.

## MATERIALS AND METHODS

### Plasma samples

Plasma samples from HCV-positive donors representing genotypes 1a, 1b, 2b, and 3a were obtained from SeraCare (Milford, MA). HIV-1 subtype B, consisting of culture supernatant diluted in defibrinated plasma, was obtained from the Virology Quality Assurance Program at the Duke Human Vaccine Institute. Basematrix Negative Diluent (SeraCare), a human plasma-derived diluent, was used for sample dilution.

### 8E5 DNA

Cells were obtained through the NIH HIV Reagent Program, Division of AIDS, NIAID, NIH: HIV-1 Lymphadenopathy-Associated Virus-infected 8E5 Cells, ARP-110, contributed by Dr. Thomas Folks. Genomic DNA was extracted from 8E5 cells using the Quick-DNA Microprep Kit (Zymo Research, Irvine, CA). Purified genomic DNA was quantified using the Qubit 1X dsDNA HS Assay Kit (Thermo Fisher Scientific, Waltham, MA). HIV-1 proviral copy numbers were estimated from genomic DNA mass assuming one proviral copy per diploid 8E5 cell and a haploid human genome mass of 3.3 pg; these estimates were used for approximate input normalization.

### Contrived whole-blood preparation

Donor whole blood collected in BD Vacutainer EDTA tubes (BD, Franklin Lakes, NJ) was centrifuged on the day of collection at 520 × *g* for 13 min to separate plasma from the RBC and buffy coat fractions. The RBC and buffy coat fractions were isolated, mixed by inversion, and recombined with viral plasma reference material at a 1:1 (vol/vol) ratio to generate contrived whole blood samples, which were used for experiments on the same day. To obtain a range of viral loads, plasma reference material was serially diluted using Basematrix Negative Diluent (SeraCare) prior to the addition of RBCs and buffy coat. For negative extraction controls, RBCs and buffy coat were added to equal volumes of Basematrix Negative Diluent.

### Viral RNA extraction

Purified viral RNA was used as template material for RT-LAMP optimization and CRISPR-Cas12a assay development. HCV genotype 1a-positive plasma and HIV-1 subtype B-positive plasma reference material were extracted using the Quick-RNA Viral Column Kit (Zymo Research, Irvine, CA). RNA extractions were performed according to the manufacturer’s protocol with the addition of a 10% proteinase K treatment. For each extraction, 200 µL of plasma was used as input. Purified RNA was eluted in nuclease-free water (Thermo Fisher Scientific, Waltham, MA), pooled, serially diluted in nuclease-free water, and aliquoted. Viral RNA concentrations were estimated based on the vendor-reported plasma viral load, extraction input volume, elution volume, and the assumption of negligible RNA loss during extraction and elution. RNA extracts were stored at −80°C until use.

### RT-LAMP primers

Previously published RT-LAMP primer sets (Table S1) were selected based on their *in silico* design targeting highly conserved genomic regions across multiple viral subtypes or genotypes of regional clinical relevance in Canada, as well as prior demonstration of detecting HIV-1 subtype B and HCV genotype 1a in extracted RNA from clinical samples. Tenfold (10×) primer mixes contained the following final primer concentrations: 16 µM forward inner primer and backward inner primer, 8 µM loop forward and loop backward, and 2 µM forward outer primer (F3) and backward outer primer (B3). To enable lateral-flow dipstick detection, the 10× primer mixes used for the extraction-free RT-LAMP workflow included primers labeled with biotin and 6-carboxyfluorescein (FAM). All primers were synthesized with standard desalting and lyophilized (Integrated DNA Technologies, Coralville, IA).

### Off-the-shelf lyophilized RT-LAMP

A lyophilized RT-LAMP reagent, LyoPrime WarmStart Fluorescent LAMP/RT-LAMP Mix (with UDG) (New England Biolabs, Ipswich, MA), was rehydrated with nuclease-free water according to the manufacturer’s instructions. Each optimized 25 µL RT-LAMP reaction contained 12.5 µL of rehydrated RT-LAMP mix, 2.5 µL of nuclease-free water, 2.5 µL of 10× primer mix, betaine at a final concentration of 0.5 M (Sigma-Aldrich, St. Louis, MO), and 5 µL of template. Primer screening and evaluation of the effects of blood diluents and dilution ratios on extraction-free HCV RT-LAMP were conducted at an incubation temperature of 62°C for 60 min with a final betaine concentration of 0.5 M. For extraction-free HCV and HIV-1 RT-LAMP LLOD experiments, reactions were incubated for 30 min under specific temperature and betaine concentration profiles outlined in Tables 2 and 3. RT-LAMP reactions were performed using either the QuantStudio 12K Flex or QuantStudio 5 Real-Time PCR System (Thermo Fisher Scientific).

### Cold-stored RT-LAMP

RT-LAMP reactions were assembled in a final volume of 25 µL. Each reaction contained 2.5 µL of 10× Isothermal Amplification Buffer (New England Biolabs), which includes magnesium sulfate, 3.5 µL of dNTP mix (10 mM each), 2.5 µL of SYTO-9 fluorescent dye (5 µM), 0.5 µL of WarmStart RTx Reverse Transcriptase (15 U/µL), and 0.067 µL of Bst 2.0 DNA Polymerase (120 U/µL). Betaine was added from a 5 M stock (Sigma-Aldrich) to a final concentration of 0 or 0.5 M. Additional magnesium sulfate was supplemented from a 100 mM stock solution to achieve final concentrations of 4.5 mM, 6.0 mM, or 7.0 mM, depending on the experimental condition. Template input volume was 5 µL. Nuclease-free water was added to bring the final reaction volume to 25 µL after addition of all components.

### Synthetic templates for RT-LAMP

Synthetic gBlock Gene Fragments (Integrated DNA Technologies), containing pathogen-specific target sequences (Table S12), were used as templates for positive controls in RT-LAMP experiments.

### Real-time detection

Data analysis was performed using Design & Analysis Software v.2.6.0 or QuantStudio 12K Flex Software v.1.4 (Thermo Fisher Scientific), using the ∆Rn versus cycle number plot. For determination of the LLOD, the ∆Rn fluorescence threshold was set at 2,500,000. The TTP, defined as the time at which the amplification curve crossed the fluorescence threshold, was calculated for each condition and rounded to the nearest whole minute. An input amount was designated as the LLOD based on two criteria: (i) the LLOD corresponded to the lowest input amount at which all replicates exhibited both a measurable TTP and a positive CRISPR-Cas12a detection; (ii) all replicates at input amounts above the LLOD were required to also exhibit a measurable TTP and positive CRISPR-Cas12a detection. Criterion (ii) was not applied if the LLOD represented the highest input amount tested.

### Evaluation of blood diluents and dilution ratios

A contrived whole blood sample positive for HCV genotype 1a was diluted at 1:1 or 1:3 ratios (one part sample to one or three parts diluent) using nuclease-free water, ACK lysing buffer (Thermo Fisher Scientific), or red blood cell lysis buffer (18). For each condition, 5 µL of diluted or undiluted contrived whole blood was added directly to 20 µL of HCV RT-LAMP master mix. Reactions were performed in triplicate and incubated at 62°C for 60 min using the QuantStudio 12K Flex Real-Time PCR System.

### CRISPR-Cas12a detection

CRISPR-Cas12a assays were performed to verify RT-LAMP target amplicons. Target-specific guide RNAs were designed using the CHOPCHOP online tool (54). All guide RNAs were synthesized with standard desalting (Integrated DNA Technologies). Cas12a ribonucleoprotein complexes were prepared as previously described (36), with the modification that guide RNAs were used at a final concentration of 100 nM and applied in trans-cleavage reactions. Each 100 µL reaction contained 18 µL of RNP complex, 0.5 µM single-stranded DNA reporter (/5YakYel/TTATTATT/3IABkFQ/) (Integrated DNA Technologies), 0.75× NEBuffer r2.1 (New England Biolabs), and 2 µL of RT-LAMP product. HCV guide RNA 1 and HIV-1 guide RNA 1 (Table S5) were used unless otherwise noted. Reactions were incubated at 37°C for 15 min using the QuantStudio 12K Flex Real-Time PCR System. Real-time fluorescence was measured using the VIC reporter channel and analyzed using the multicomponent plot in the QuantStudio 12K Flex Software v.1.4.

### Lateral-flow readout

Following RT-LAMP, 30 µL of nuclease-free water was added to each reaction prior to immersion of a Milenia HybriDetect Universal lateral-flow dipstick (TwistDx, Cambridge, UK). After 2 min, dipsticks were imaged using a M200 camera (Canon Inc., Tokyo, Japan). Positive results were defined by the presence of both a control band (upper band) and a test band (lower band), whereas the presence of the control band alone indicated a negative result. Lateral-flow readout results were used to determine the LLOD for extraction-free HIV-1 RT-LAMP and extraction-free HCV RT-LAMP experiments. An input amount was designated as the LLOD based on two criteria: (i) the lowest input amount at which all replicates exhibited a positive lateral flow result, and (ii) positive lateral flow results for all replicates at input amounts above the LLOD.

### Viral load quantification and estimation

Viral loads of contrived HCV whole blood samples were quantified using the Xpert HCV VL Fingerstick assay (Cepheid, Sunnyvale, CA), while HIV-1 viral loads were estimated using the Xpert HIV-1 Qual assay (Cepheid, Sunnyvale, CA), following the manufacturer’s instructions. HIV-1 viral loads were estimated by back-calculating from the cycle-threshold values of the Xpert HIV-1 Qual (93 min runtime) via the linear regression equation provided in the manufacturer’s instructions for use.

### Portable dual-pathogen diagnostic workflow

To demonstrate assay performance under portable operating conditions, the optimized extraction-free workflow was implemented using a portable heating device (Genie III; OptiGene, Horsham, UK) and without cold-chain storage. Reagents and materials stored at room temperature for at least six days were used, including lyophilized pathogen-specific 10× primer mixes containing all six primers, LyoPrime WarmStart Fluorescent LAMP/RT-LAMP Mix (with UDG), nuclease-free water, betaine, and Milenia HybriDetect Universal lateral-flow dipstick. Reactions were incubated at 62°C for 30 min. Following amplification, target amplicons were detected by lateral-flow dipstick readout and CRISPR-Cas12a fluorescence detection.

### Sequence alignment analysis

The HCV-K12 RT-LAMP primer set (21) was aligned to reference sequences representing multiple HCV genotypes (Table S10) using Sequencher software (Gene Codes Corporation, Ann Arbor, MI).

## Supplementary Material

Reviewer comments

## SUPPLEMENTAL MATERIAL

Supplemental figures and tables spectrum.00416-26-s0001.docx Figures S1 to S3 and Tables S1 to S12.

OPEN PEER REVIEW reviewer-comments.pdf An accounting of the reviewer comments and feedback.

ASM does not own the copyrights to Supplemental Material that may be linked to, or accessed through, an article. The authors have granted ASM a non-exclusive, world-wide license to publish the Supplemental Material files. Please contact the corresponding author directly for reuse.
