# Supplementary material for: Rapid, low-cost, and portable molecular detection of HIV-1 subtype B and HCV 1a directly from blood
Source: Microbiol Spectr. 2026 Aug 5;14(9):e00416-26. doi: 10.1128/spectrum.00416-26 (PMC13532246; doi:10.1128/spectrum.00416-26)
Supplement: Supplementary file 1 [file reviewer-comments.pdf]

AMERICAN SOCIETY FOR MICROBIOLOGY | Microbiology Spectrum

Rapid, Low-Cost and Portable Molecular Detection of HIV-1 Subtype B and HCV 1a Directly from Blood

David Tang, Karen Michalak, Kelvin Nguyen, Philip Lacap, John Kim, and Paul McLaren

Corresponding Author(s): David Tang, Public Health Agency of Canada

Review Timeline:

Submission Date:	February 5, 2026
Editorial Decision:	March 26, 2026
Revision Received:	May 26, 2026
Accepted:	June 11, 2026

Editor: Kenneth Gavina

Reviewer(s): The reviewers have opted to remain anonymous.

Transaction Report:

(Note: With the exception of the correction of typographical or spelling errors that could be a source of ambiguity, letters and reports are not edited. The original formatting of letters and referee reports may not be reflected in this compilation.)

DOI: https://doi.org/10.1128/spectrum.00416-26

Re: Spectrum00416-26 (Rapid, Low-Cost and Portable Molecular Detection of HIV-1 and HCV Directly from Contrived Blood)

Dear Dr. David Tang:

Thank you for the privilege of reviewing your work. Your manuscript has been reviewed by experts in the field and have been selected for modifications. Below you will find the instructions from the Spectrum editorial office and the reviewer comments. Please ensure to respond to the reviewer comments in their entirety.

Please return the manuscript within 60 days; if you cannot complete the modification within this time period, please contact me. If you do not wish to modify the manuscript and prefer to submit it to another journal, notify me immediately so that the manuscript may be formally withdrawn from consideration by Spectrum.

Revision Guidelines
To submit your modified manuscript, log into the submission site at https://spectrum.msubmit.net/cgi-bin/main.plex. Go to Author Tasks and click the appropriate manuscript title to begin. The information you entered when you first submitted the paper will be displayed; update this as necessary. Note the following requirements:

• Upload point-by-point responses to the issues raised by the reviewers in a file named "Response to Reviewers," NOT in your cover letter.
• Upload a compare copy of the manuscript (without figures) as a "Marked-Up Manuscript" file.
• Upload a clean .DOC/.DOCX version of the revised manuscript and remove the previous version.
• Each figure must be uploaded as a separate, editable, high-resolution file (TIFF or EPS preferred), and any multipanel figures must be assembled into one file.
• Any supplemental material intended for posting by ASM should be uploaded with their legends separate from the main manuscript. You can combine all supplemental material into one file (preferred) or split it into a maximum of 10 files with all associated legends included.

For complete guidelines on revision requirements, see our Submission and Review Process webpage. Submission of a paper that does not conform to guidelines may delay acceptance of your manuscript.

Data availability: ASM policy requires that data be available to the public upon online posting of the article, so please verify all links to sequence records, if present, and make sure that each number retrieves the full record of the data. If a new accession number is not linked or a link is broken, provide Spectrum production staff with the correct URL for the record. If the accession numbers for new data are not publicly accessible before the expected online posting of the article, publication may be delayed; please contact production staff (Spectrum@asmusa.org) immediately with the expected release date.

Publication Fees: For information on publication fees and which article types are subject to charges, visit our website. If your manuscript is accepted for publication and any fees apply, you will be contacted separately about payment during the production process; please follow the instructions in that e-mail. Arrangements for payment must be made before your article is published.

ASM Membership: Corresponding authors may join or renew ASM membership to obtain discounts on publication fees. Need to upgrade your membership level? Please contact Customer Service at Service@asmusa.org.

The ASM Journals program strives for constant improvement in our submission and publication process. Please tell us how we can improve your experience by taking this quick Author Survey.

Thank you for submitting your paper to Spectrum.

Sincerely,
Kenneth Gavina
Editor
Microbiology Spectrum

Reviewer #1 (Comments for the Author):

"Rapid, Low-Cost and Portable Molecular Detection of HIV-1
and HCV Directly from Contrived Blood"

In this manuscript by Tang et al. the authors outline the development of a proof-of-concept molecular-based assay for the detection of HIV-1 and HCV. The main findings of the study provide performance data to demonstrate the overall feasibility of

the proposed testing workflow. Admirably, the authors sought to develop a rapid molecular-diagnostic that overcomes many cited limitations of existing molecular assays for these pathogens, namely, a need for cold-stored reagents, plug-in power, and/or complex amplicon detection approaches. Although significant additional work would be required to achieve regulatory approval as a medical assay, this study provides an opportunity for further investigations to build upon. Moreover, the study adequately frames the work as 'proof-of-concept'. I would suggest the following considerations be addressed to strengthen the manuscript.

Major comments
• The possibility of non-specific LAMP products is of particular concern. Lines 134-136 indicate that non-specific reactions occur at or greater than 54 minutes, referring readers to Supplementary Tables 2 and 5-8. Table S2 (last row) shows that, with betaine, none of the amplifications were positive, that the average time to positive (which were all false positives) was 42 minutes and that the range was 21-57 minutes. Therefore, the statement in the results section lines 134-136 seem exaggerated and need to be clarified. In addition, I think it would be of benefit for the readers to see these particular results (last two rows of Table S2) presented as a scatter plot with each individual data points shown in the figure, so that readers can fully appreciate the time trajectory of false positives.
• Lines 70-71: The authors claim an urgent need for "...affordable..." testing. The provision of costing information for the proposed RT-LAMP with dipstick reporting would help support the notion that the proof-of-concept approach is cost-effective.
• Section 3.4 seems unnecessary. It adds text (20 lines) and figure/tables (1 figure + 2 tables in the supplement) to an already lengthy study and the findings do not appreciably add to the main study results. Furthermore, this reviewer is confused why the in silico analysis to confirm suitability of the HCV primers for detection of HCV genotypes 2b and 3a would not have been performed up-front, prior to experimentation. Without section 3.4, I imagine that some readers will wonder about the performance of the developed HCV RT-LAMP to detect the array of HCV circulating genotypes other than genotype 1b; however, this could be satisfied with a very limited description in the discussion section (data not shown).

Minor comments
• Line 142/145: please clarify what is meant by "pathogenic blood samples", consider revising to better explain.
• Supplemental Tables 6 & 7: the table legends of both tables begins with "Values bolded in green indicate the lower limit of detection (LLOD) of each HIV-1 RTLAMP condition". It would be helpful to readers if the authors could be more explicit, stating that LLODs were determined for each incubation temp-betain conc permutation.
• Supplemental Tables 6 & 7: footnote 'B' for both tables pertains to the column heading "RT-LAMP Condition". The footnote describes that the "reactions were incubated at 62C for 60 minutes"; however, the column then provides separate lines of data for incubation temperatures of 61, 62, and 63C. The footnote could be written more generically to indicate that reactions were incubated at the 'indicated' temperature for 60 minutes.
• Lines 104-106: it would seem as though the font is different
• Line 122: Was the source of the "off-the-shelf" master mix ever stated? If not, is there a reason why?
• Supplemental Table 6: footnote B starts with "Off-shelf...", should this be "Off-the-shelf..."?

Reviewer #2 (Comments for the Author):

The authors describe a portable and rapid molecular test for detection of HIV and HCV RNA. Importantly, evaluation involves testing dilution strategies to decrease the turn around time and increase sensitivity. Suboptimal LLOD is nicely explained in the context of proposed clinical and setting use. Overall, the study is justified as this type of technology is needed and test is promising but I have some suggestions:
1. The title is not very clear. Suggest something like "A rapid and portable molecular test for detection of HIV-1 and HCV 1a directly from blood"
a. In practice, the idea is you wouldn't use contrived blood and although this is what was used in the study for evaluation it's not the purpose
b. I would remove cost. while the assumption of low cost can be mentioned in the introduction and discussion, the authors do not present data on test cost (unless this is something the authors can add in)
c. The assay only detected certain genotypes of HCV and title should reflect that
2. Line 103: Although RT-LAMP has been applied to HIV-1 and HCV detection, its use in MPOC settings remains limited. do you mean its use in resource limited settings? Or do you mean its use in MPOC tests? Unclear.
3. Specificity analyses: the authors present a method to determine if positive test results represent non-target amplicons, which is great. however, they do not test the assay against other closely related or common pathogens to ensure that n false-positive signals occur.
4. Authors should mention this is a qualitative test
5. The authors state they we sought to develop and evaluate a test for detection of HCV genotype 1a and HIV-1 subtype B. However other HCV genotypes were used but the test did not work well against other reference strains used although 3s was detected.
6. A major limitation of this study is that only certain reference strains were used to evaluate this assay, namely AF033819.3 (HIV-1 subtype B) and AF009606.1 (HCV 1a) but also strains representing other HCV genotypes. It is not stated in the study if the reference strains chosen represent contemporary strains/all strains of the subtype or if there is diversity among subtypes and the test should be tested against other strains. If the latter is the case, the authors should use other strains in their evaluation to

better understand the performance of the test for HIV and HCV broadly. Or authors could clearly state that the purpose of the study is a proof of principal to see if RNA can be detected and that future studies should evaluate various strain types (if relevant) as well as optimize primers as needed (as evidenced by mismatches).

7. Line 214 authors mention a technical error. If a known technical error occurred why was the test not repeated?

8. Access to testing is one piece of the puzzles. The authors should consider adding a sentence to discussion that relates to the need for comprehensive healthcare services that include links to public health, treatment and care.

“Rapid, Low-Cost and Portable Molecular Detection of HIV-1 and HCV Directly from Contrived Blood”

In this manuscript by Tang et al. the authors outline the development of a proof-of-concept molecular-based assay for the detection of HIV-1 and HCV. The main findings of the study provide performance data to demonstrate the overall feasibility of the proposed testing workflow. Admirably, the authors sought to develop a rapid molecular-diagnostic that overcomes many cited limitations of existing molecular assays for these pathogens, namely, a need for cold-stored reagents, plug-in power, and/or complex amplicon detection approaches. Although significant additional work would be required to achieve regulatory approval as a medical assay, this study provides an opportunity for further investigations to build upon. Moreover, the study adequately frames the work as ‘proof-of-concept’. I would suggest the following considerations be addressed to strengthen the manuscript.

Major comments

- The possibility of non-specific LAMP products is of particular concern. Lines 134-136 indicate that non-specific reactions occur at or greater than 54 minutes, referring readers to Supplementary Tables 2 and 5-8. Table S2 (last row) shows that, with betaine, none of the amplifications were positive, that the average time to positive (which were all false positives) was 42 minutes and that the range was 21-57 minutes. Therefore, the statement in the results section lines 134-136 seem exaggerated and need to be clarified. In addition, I think it would be of benefit for the readers to see these particular results (last two rows of Table S2) presented as a scatter plot with each individual data points shown in the figure, so that readers can fully appreciate the time trajectory of false positives.
- Lines 70-71: The authors claim an urgent need for “…affordable…” testing. The provision of costing information for the proposed RT-LAMP would help support the notion that the proof-of-concept approach is cost-effective.
- Section 3.4 seems unnecessary. It adds text (20 lines) and figure/tables (1 figure + 2 tables in the supplement) to an already lengthy study and the findings do not appreciably add to the main study results. Furthermore, this reviewer is confused why the *in silico* analysis to confirm suitability of the HCV primers for detection of HCV genotypes 2b and 3a would not have been performed up-front, prior to experimentation. Without section 3.4, I imagine that some readers will wonder about the performance of the developed HCV RT-LAMP to detect the array of HCV circulating genotypes other than genotype 1b; however, this could be satisfied with a very limited description in the discussion section (data not shown).

Minor comments

- Line 142/145: please clarify what is meant by “pathogenic blood samples”, consider revising to better explain.
- Supplemental Tables 6 & 7: the table legends of both tables begins with “Values bolded in green indicate the lower limit of detection (LLOD) of each HIV-1 RTLAMP condition”. It would be helpful to readers if the authors could be more explicit, stating that LLODs were determined for each incubation temp-betain conc permutation.

- Supplemental Tables 6 & 7: footnote ‘B’ for both tables pertains to the column heading “RT-LAMP Condition”. The footnote describes that the “reactions were incubated at 62°C for 60 minutes”; however, the column then provides separate lines of data for incubation temperatures of 61, 62, and 63°C. The footnote could be written more generically to indicate that reactions were incubated at the ‘indicated’ temperature for 60 minutes.
- Lines 104-106: it would seem as though the font is different
- Line 122: Was the source of the “off-the-shelf” master mix ever stated? If not, is there a reason why?
- Supplemental Table 6: footnote B starts with “Off-shelf…”, should this be “Off-the-shelf…”?

<u>Reviewer #1 (Comments for the Author):</u>
"Rapid, Low-Cost and Portable Molecular Detection of HIV-1 and HCV Directly from Contrived Blood"

In this manuscript by Tang et al. the authors outline the development of a proof-of-concept molecular-based assay for the detection of HIV-1 and HCV. The main findings of the study provide performance data to demonstrate the overall feasibility of the proposed testing workflow. Admirably, the authors sought to develop a rapid molecular-diagnostic that overcomes many cited limitations of existing molecular assays for these pathogens, namely, a need for cold-stored reagents, plug-in power, and/or complex amplicon detection approaches. Although significant additional work would be required to achieve regulatory approval as a medical assay, this study provides an opportunity for further investigations to build upon. Moreover, the study adequately frames the work as 'proof-of-concept'. I would suggest the following considerations be addressed to strengthen the manuscript.

<u>Review 1, major comment 1:</u>
The possibility of non-specific LAMP products is of particular concern. Lines 134-136 indicate that non-specific reactions occur at or greater than 54 minutes, referring readers to Supplementary Tables 2 and 5-8. Table S2 (last row) shows that, with betaine, none of the amplifications were positive, that the average time to positive (which were all false positives) was 42 minutes and that the range was 21-57 minutes. Therefore, the statement in the results section lines 134-136 seem exaggerated and need to be clarified.

In addition, I think it would be of benefit for the readers to see these particular results (last two rows of Table S2) presented as a scatter plot with each individual data points shown in the figure, so that readers can fully appreciate the time trajectory of false positives.

Author's reply:
We thank the reviewer for this observation. We acknowledge that the original text did not sufficiently distinguish between the results generated using a cold-stored custom master mix (Supplementary Tables 2 and 4) and the off-the-shelf lyophilized master mix (Supplementary Tables 6 and 7). The data in Supplementary Table 2 was generated using a cold-stored master mix with deliberately elevated magnesium sulphate concentrations. This was done to induce non-target amplicons and demonstrate the specificity and utility of our custom-designed CRISPR-Cas12 guide RNAs. To resolve any potential confusion, we have reordered the supplementary tables and updated the corresponding citations within the manuscript to ensure technical clarity and consistency. We have revised the manuscript to clarify that the time-to-positive (TTP)

delay for non-target amplicons refers specifically to results obtained with the off-the-shelf lyophilized master mix. Additionally, we have corrected a typographical error in the original text, updating the TTP from ≥54 minutes to ≥55 minutes.

Furthermore, we agree that a scatter plot showing individual TTP values for no-template controls in HCV RT-LAMP reactions under variable betaine concentrations helps the reader appreciate the distribution of false positives.

Manuscript revision:

Line 132 (revised): "Given the tendency of RT-LAMP to generate non-target amplicons depending on the master mix formulation (Supplementary Figure 1; Supplementary Tables 2 and 4; Kim et al., 2023; Meagher et al., 2018), we incorporated CRISPR-Cas12 fluorescence detection with custom-designed guide RNAs (Supplementary Tables 5 and 6)…"

Line 138 (revised): "This approach revealed that when using the off-the-shelf lyophilized master mix, non-target amplicons occurred very infrequently and exhibited delayed TTPs (≥55 minutes) compared to target amplicons (Supplementary Tables 7 and 8)."

Added a reference to Supplementary Figure 1 at Line 132 and included the figure in the Supplementary Information.

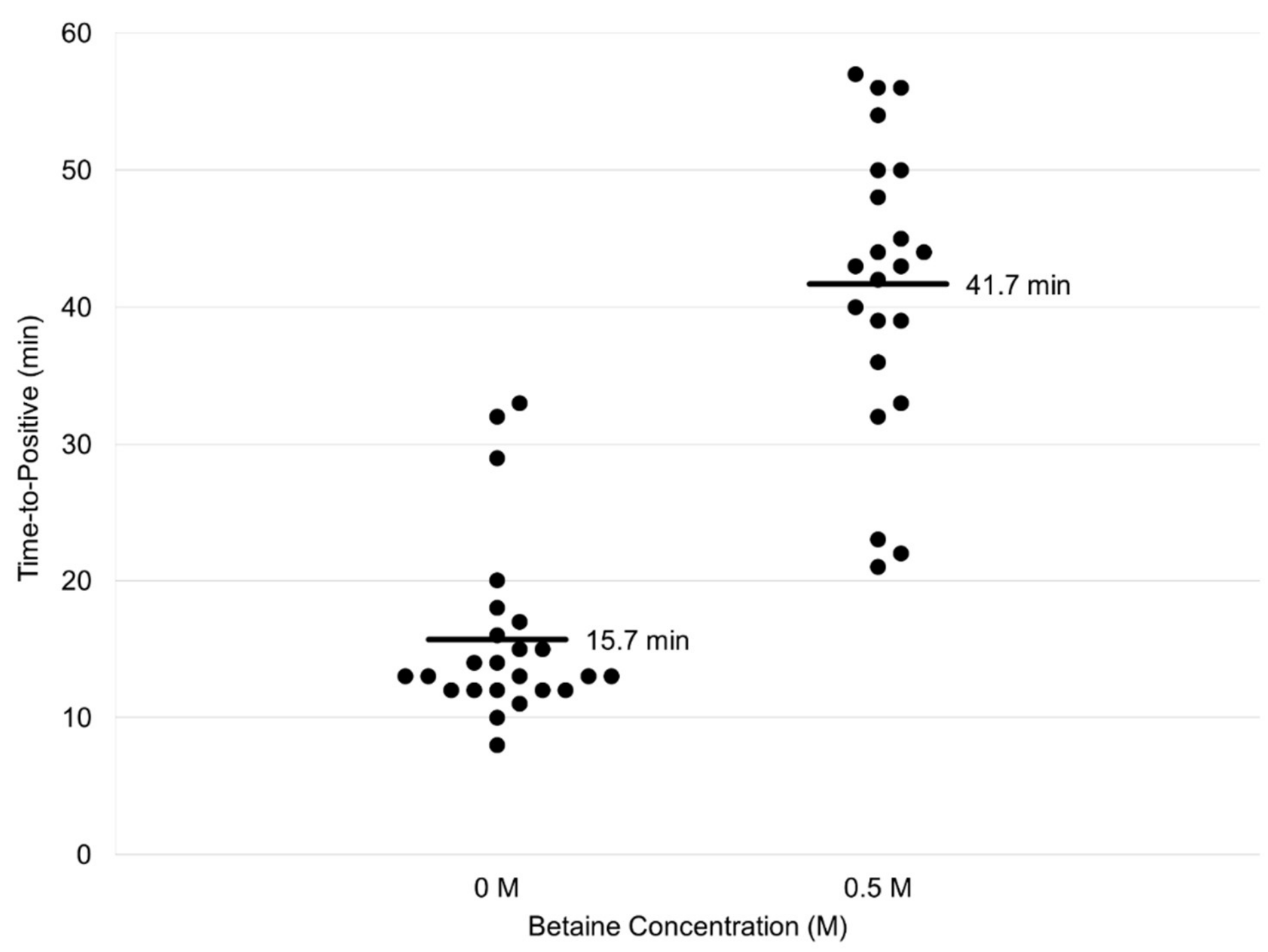

Supplementary Figure 1. Distribution of time-to-positive (TTP) values for non-target amplicons in no-template controls from HCV RT-LAMP reactions under variable betaine concentrations. Individual TTP values and CRISPR detection results

are shown for replicates described in Supplementary Table 2. These reactions utilized a cold-stored master mix with elevated magnesium (6 mM) to deliberately promote non-specific amplification in no-template control reactions. For the 0 M betaine group (n=24), all replicates exhibited non-target amplification. For the 0.5 M betaine group, 20 of 22 technical replicates (n=22) exhibited a TTP within the 60-minute runtime; the two replicates that showed no amplification were not plotted as they lack a TTP value. Horizontal bars and adjacent numerical labels represent the mean TTP of the amplifying replicates within each group (15.7 min for 0 M betaine and 41.7 min for 0.5 M betaine). These data illustrate that the addition of 0.5 M betaine not only reduces the frequency of non-target amplicons but also significantly delays their time trajectory, providing greater separation between TTP values of target and non-target amplification.

Reviewer 1, major comment 2:
Lines 70-71: The authors claim an urgent need for "...affordable..." testing. The provision of costing information for the proposed RT-LAMP with dipstick reporting would help support the notion that the proof-of-concept approach is cost-effective.

Author's reply:
We thank the reviewer for this suggestion. We agree that providing cost information will help the reader understand the cost-effectiveness per test. We have added an estimated per-test consumable cost to the Discussion section.

Manuscript revision:

Line 400 (revised). "Based on current research-use pricing, the consumable cost per test is approximately $7 USD, including the lyophilized RT-LAMP reaction, primer set, betaine, and lateral flow dipstick, and excluding capital equipment and control reactions."

Reviewer 1, major comment 3:
Section 3.4 seems unnecessary. It adds text (20 lines) and figure/tables (1 figure + 2 tables in the supplement) to an already lengthy study and the findings do not appreciably add to the main study results. Furthermore, this reviewer is confused why the in silico analysis to confirm suitability of the HCV primers for detection of HCV genotypes 2b and 3a would not have been performed up-front, prior to experimentation. Without section 3.4, I imagine that some readers will wonder about the performance of the developed HCV RT-LAMP to detect the array of HCV circulating genotypes other than genotype 1b; however, this could be satisfied with a very limited description in the discussion section (data not shown).

Author's reply:

We thank the reviewer for this thoughtful comment. We agree that Section 3.4 did not add to the main results. We have removed this section from the main text and incorporated a concise summary of these findings into the discussion (lines 346 to 354), with the corresponding data referenced as Supplementary Figure 3 and Supplementary Tables 10 and 11.

We have also clarified the rationale for the in silico analysis.

Manuscript revision:

Line 346 to 354 in the Discussion section

"While genotype 1b was detected with comparable sensitivity, reduced or absent amplification was observed for genotypes 2b and 3a (Supplementary Figure 3; Supplementary Table 10). *Post-hoc* sequence alignment revealed several primer–template mismatches within those specific genotypes (Supplementary Table 11), elucidating the underlying cause of the reduced amplification efficiency. Although the HCV-K12 primer set was originally described as pan-genotypic (Kargar et al., 2012), our findings indicate that sequence-specific optimization may be necessary to achieve high sensitivity within this rapid, extraction-free, and lyophilized RT-LAMP workflow. These findings establish an analytical benchmark for the development of primer sets capable of achieving comparable or improved sensitivity (3.77 $\log_{10}$ IU/mL) across the most clinically relevant genotypes while remaining compatible with this simplified workflow. While further validation is required to evaluate the workflow as a first-line molecular screening tool in low-prevalence populations, its immediate utility lies in high-prevalence settings, where viral loads frequently exceed 6 $\log_{10}$ IU/mL (Mohamed et al., 2020)."

<u>Review 1, minor comment 1</u>

Line 142/145: please clarify what is meant by "pathogenic blood samples", consider revising to better explain

Author's reply:

We thank the reviewer for this note. We agree that the term "pathogenic blood samples" was imprecise. We have revised the manuscript at Lines 150 to 151 to use "..blood samples containing viral pathogens (HIV-1 and HCV).." to more clearly describe the samples containing HIV-1 and HCV.

<u>Review 1, minor comment 2</u>

Supplemental Tables 6 & 7: the table legends of both tables begins with "Values bolded in green indicate the lower limit of detection (LLOD) of each HIV-1 RTLAMP condition".

It would be helpful to readers if the authors could be more explicit, stating that LLODs were determined for each incubation temp-betain conc permutation.

Author's reply:

We thank the reviewer for this helpful suggestion. We agree that the original table legends could be more explicit. We revised the legends of Supplemental Tables 7 and 8 to clarify that the lower limit of detection (LLOD) was determined for each incubation temperature and betaine concentration permutation.

Review 1, minor comment 3

Supplemental Tables 6 & 7: footnote 'B' for both tables pertains to the column heading "RT-LAMP Condition". The footnote describes that the "reactions were incubated at 62C for 60 minutes"; however, the column then provides separate lines of data for incubation temperatures of 61, 62, and 63C. The footnote could be written more generically to indicate that reactions were incubated at the 'indicated' temperature for 60 minutes.

Author's reply:

We thank the reviewer for this comment. We agree that the original wording of footnote B could be interpreted as applying only to 62°C. We have revised the footnote in Supplementary Tables 7 and 8 to clarify that "reactions were incubated at the indicated temperature for 60 minutes".

Review 1, minor comment 4

Lines 104-106: it would seem as though the font is different

Author's reply:

We thank the review for this comment. We have corrected the font style in this section for consistency with the rest of the manuscript.

Review 1, minor comment 5

Line 122: Was the source of the "off-the-shelf" master mix ever stated? If not, is there a reason why?

Author's reply:

We thank the reviewer for this comment. While the specific reagent name (LyoPrime WarmStart Fluorescent LAMP/RT-LAMP Mix [with UDG]) was included in the Methods

section, we agree that the manufacturer and the "off-the-shelf" nature of the reagent should be more explicitly linked.

Manuscript revision:

In Results Section 3.1 (Line 123), we have now introduced the reagent name upon first mention: "...primer sets (Supplementary Table 1) were screened using an off-the-shelf lyophilized master mix (LyoPrime WarmStart Fluorescent LAMP/RT-LAMP Mix [with UDG]) and serially diluted extracted viral RNA."

In addition, we have revised the Methods to include the manufacturer (New England Biolabs, Ipswich, MA) alongside the product name.

Furthermore, we have updated the subheading in the Methods section to "Off-the-Shelf Lyophilized RT-LAMP" to better align with the terminology used in the results and to clarify that this is a commercially available master mix.

Review 1, minor comment 6

Supplemental Table 6: footnote B starts with "Off-shelf...", should this be "Off-the-shelf..."?

Author's reply:
We thank the reviewer for this observation. We have corrected the footnote in Supplementary Table 7 and 8 to read: "Off-the-shelf lyophilized master mix..." to ensure consistency with the terminology used throughout the text.

Reviewer #2 (Comments for the Author):

The authors describe a portable and rapid molecular test for detection of HIV and HCV RNA. Importantly, evaluation involves testing dilution strategies to decrease the turn around time and increase sensitivity. Suboptimal LLOD is nicely explained in the context of proposed clinical and setting use. Overall, the study is justified as this type of technology is needed and test is promising but I have some suggestions:

Reviewer 2, comment 1:

1. The title is not very clear. Suggest something like "A rapid and portable molecular test for detection of HIV-1]subtype B]and HCV 1a directly from blood"
a. In practice, the idea is you wouldn't use contrived blood and although this is what was used in the study for evaluation it's not the purpose
b. I would remove cost. while the assumption of low cost can be mentioned in the introduction and discussion, the authors do not present data on test cost (unless this is something the authors can add in)
c. The assay only detected certain genotypes of HCV and title should reflect that

Author's reply:
We thank the review for the suggestions. We revised the title to be "Rapid, Low-Cost, and Portable Molecular Detection of HIV-1 Subtype B and HCV Genotype 1a Directly from Blood".

We retained the term low-cost in the title because we now included an estimated per-test cost in the Discussion section. Line 400 (revised). "Based on current research-use pricing, the consumable cost per test is approximately $7 USD, including the lyophilized RT-LAMP reaction, primer set, betaine, and lateral flow dipstick, and excluding capital equipment and control reactions."

Reviewer 2, comment 2
Line 103: Although RT-LAMP has been applied to HIV-1 and HCV detection, its use in MPOC settings remains limited. do you mean its use in resource limited settings? Or do you mean its use in MPOC tests? Unclear.

Author's reply
We thank the reviewer for the careful reading. Line 103 has been revised to: "Although RT-LAMP has been applied to HIV-1 and HCV detection, its use in MPOC tests remains limited."

<u>Reviewer 2, comment 3</u>
Specificity analyses: the authors present a method to determine if positive test results represent non-target amplicons, which is great. however, they do not test the assay against other closely related or common pathogens to ensure that n false-positive signals occur.

Author's reply:
We thank the reviewer for highlighting the importance of broad analytical specificity.

The primer sets used in this study were originally designed and experimentally evaluated for analytical specificity by prior authors. In those studies, RT-LAMP reactions were performed using laboratory-prepared master mixes formulated from cold-stored reagents, rather than lyophilized reagents. The HCV-K12 primer set (Kargar et al., 2012) was designed against a conserved region of the HCV genome and was reported to detect multiple genotypes (1a, 1b, 2, and 3a), with no cross-reactivity observed for CMV, HBV, and HSV-1. Similarly, the HIV-1 primer set (Zeng et al., 2014) was designed based on a conserved region of the HIV-1 genome and demonstrated no cross-reactivity to HIV-2, HTLV-1, and HCV.

We acknowledge, however, that RT-LAMP primer performance can vary depending on reaction conditions and the master mix formulation. To partially address this in the context of our extraction-free workflow using a lyophilized master mix, we evaluated cross-reactivity using available non-target viral samples. As described in the Results (line 274), "assay specificity was further assessed by demonstrating that the extraction-free HCV RT-LAMP assay did not generate target amplicons from HIV-1 subtype B-containing samples, and conversely, the extraction-free HIV-1 RT-LAMP assay did not generate target amplicons from HCV genotype 1a-containing samples (Supplementary Figure 2)."

We recognize that this limited evaluation does not fully address potential cross-reactivity with other clinically relevant pathogens; broader specificity testing will be required in future studies as this proof-of-concept workflow is further developed.

<u>Reviewer 2, comment 4</u>
Authors should mention this is a qualitative test

Author's reply:

We thank the reviewer for this suggestion. We agree that defining the assay's intended use as qualitative is important for clinical clarity.

Manuscript revisions:

Abstract, line 22:

…" simultaneous qualitative detection of.."

Introduction, line 112:
"In this study, we sought to develop and analytically evaluate a 32-minute total-runtime, portable, low-cost, proof-of-concept molecular diagnostic workflow for the simultaneous qualitative detection of HCV genotype 1a and HIV-1 subtype B directly from contrived whole blood."

Reviewer 2, comment 5
The authors state they we sought to develop and evaluate a test for detection of HCV genotype 1a and HIV-1 subtype B. However other HCV genotypes were used but the test did not work well against other reference strains used although 3s was detected.

Author's reply:

We thank the reviewer for this observation, which was also raised by Reviewer 1 (Major Comment 3). We agree that the performance across other HCV genotypes was variable compared to our primary target (genotype 1a). As suggested by Reviewer 1, we have moved the detailed genotype results (formerly Section 3.4) to the Discussion section and provided a technical interpretation of these findings, framing them as a benchmark for future primer optimization.

Manuscript revision (Discussion, Line 346)

"While genotype 1b was detected with comparable sensitivity, reduced or absent amplification was observed for genotypes 2b and 3a (Supplementary Figure 3; Supplementary Table 10). *Post-hoc* sequence alignment revealed several primer–template mismatches within those specific genotypes (Supplementary Table 11), elucidating the underlying cause of the reduced amplification efficiency. Although the HCV-K12 primer set was originally described as pan-genotypic (Kargar et al., 2012), our findings indicate that sequence-specific optimization may be necessary to achieve high sensitivity within this rapid, extraction-free, and lyophilized RT-LAMP workflow. These findings establish an analytical benchmark for the development of primer sets capable of achieving comparable or improved sensitivity (3.77 $\log_{10}$ IU/mL) across the most clinically relevant genotypes while remaining compatible with this simplified workflow. While further validation is required to evaluate the workflow as a first-line molecular

screening tool in low-prevalence populations, its immediate utility lies in high-prevalence settings, where viral loads frequently exceed 6 $\log_{10}$ IU/mL (Mohamed et al., 2020)."

Reviewer 2, comment 6

A major limitation of this study is that only certain reference strains were used to evaluate this assay, namely AF033819.3 (HIV-1 subtype B) and AF009606.1 (HCV 1a) but also strains representing other HCV genotypes. It is not stated in the study if the reference strains chosen represent contemporary strains/all strains of the subtype or if there is diversity among subtypes and the test should be tested against other strains. If the latter is the case, the authors should use other strains in their evaluation to better understand the performance of the test for HIV and HCV broadly. Or authors could clearly state that the purpose of the study is a proof of principal to see if RNA can be detected and that future studies should evaluate various strain types (if relevant) as well as optimize primers as needed (as evidenced by mismatches).

Author's reply:

We thank the reviewer for the suggestions. We agree that our study is a proof-of-concept evaluation of the extraction-free RT-LAMP workflow and that further evaluation on strains relevant to the target region and or optimize primers is required.

We would like to clarify that the sequences AF033819.3 (HIV-1 subtype B) and AF009606.1 (HCV genotype 1a) were utilized as *in silico* reference templates for CRISPR guide RNA design. In contrast, the experiments to determine the lower limit of detection and demonstration of portability were performed using contemporary clinical reference materials. As described in the Methods, contrived whole-blood samples were prepared using donor plasma representing HCV genotypes 1a, 1b, 2b, and 3a (SeraCare) and HIV-1 subtype B, consisting of culture supernatant diluted in defibrinated plasma (Virology Quality Assurance Program at the Duke Human Vaccine Institute).

The manuscript has been revised to clarify the rationale for selecting the primer sets evaluated for detection sensitivity and speed. In Methods section, under the "RT-LAMP Primers" heading, we now state: "Previously published RT-LAMP primer sets (Supplementary Table 1) were selected based on their *in silico* design targeting highly conserved genomic regions across multiple viral subtypes or genotypes of regional clinical relevance in Canada, as well as prior demonstration of detecting HIV-1 subtype B and HCV genotype 1a in extracted RNA from clinical samples."

<u>Reviewer 2, comment 7</u>

Line 214 authors mention a technical error. If a known technical error occurred why was the test not repeated?

Author's reply:
We thank the reviewer for requesting clarification. We agree our explanation was not clear.

Manuscript revision: (results, 3.3, lines 207 to 210)
"While independent experimental runs yielded completely consistent results for the primary lateral-flow readout, an inadvertent discrepancy in the downstream fluorescence reporter molecule restricted the corresponding confirmatory CRISPR–Cas12a validation data to a single representative dataset"

Our clarification to the reviewer:
The extraction-free HIV-1 RT-LAMP LLOD experiment was conducted across two separate experimental days. During one of these runs, an incorrect fluorescence reporter molecule was inadvertently used in the CRISPR–Cas12a detection step following RT-LAMP amplification. As lateral flow detection had already been completed and the reaction vial discarded before this error was identified, it was not possible to repeat the CRISPR-Cas12a fluorescence detection for that specific run.

We did not repeat the full LLOD experiment for the following reasons. First, lateral-flow dipstick results provided consistent confirmation of target amplicons across all replicates on both experimental days, and this remains the primary qualitative detection method in our assay. Second, valid CRISPR–Cas12a fluorescence results were successfully obtained for the other experimental run. Third, the specificity of CRISPR–Cas12a detection using HIV-1 guide RNA 1 is further supported by data from extracted RNA (Supplementary Tables 4, 6, and 8) and by the portability experiment (Figure 4 and Supplementary Figure 2), all of which demonstrate consistent and reliable detection of target amplicons in blood samples. Collectively, these results support the validity of the reported LLOD and the overall conclusions of the study, and we are confident that this isolated technical error does not compromise the integrity of our findings.

<u>Reviewer 2, comment 8</u>
Access to testing is one piece of the puzzles. The authors should consider adding a sentence to discussion that relates to the need for comprehensive healthcare services

that include links to public health, treatment and care.

Author's reply:
We thank the reviewer for this suggestion. We included your suggestion.

Manuscript revision (discussion, lines 402 to 405):
"While lowering technical barriers and cost are essential for expanding testing access, the practical impact of this diagnostic workflow is best realized through its integration into a robust continuum of care that links results with immediate therapeutic intervention and longitudinal clinical management."

Re: Spectrum00416-26R1 (Rapid, Low-Cost and Portable Molecular Detection of HIV-1 Subtype B and HCV 1a Directly from Blood)

Dear Dr. David Tang:

I am delighted to share that your manuscript has been accepted, and I am forwarding it to the ASM production staff for publication. Your paper will first be checked to make sure all elements meet the technical requirements. ASM staff will contact you if anything needs to be revised before copyediting and production can begin. Otherwise, you will be notified when your proofs are ready to be viewed.

Data Availability: ASM policy requires that data be available to the public upon online posting of the article, so please verify all links to sequence records, if present, and make sure that each number retrieves the full record of the data. If a new accession number is not linked or a link is broken, provide production staff with the correct URL for the record. If the accession numbers for new data are not publicly accessible before the expected online posting of the article, publication may be delayed; please contact ASM production staff immediately with the expected release date.

Publication Fees: For information on publication fees and which article types have charges, please visit our website. We have partnered with Copyright Clearance Center (CCC) to collect author charges. If fees apply to your paper, you will receive a message from no-reply@copyright.com with further instructions. For questions related to paying charges through RightsLink, please contact CCC at ASM_Support@copyright.com or toll free at +1-877-622-5543. CCC makes every attempt to respond to all emails within 24 hours.

ASM Membership: Corresponding authors may join or renew ASM membership to obtain discounts on publication fees. Need to upgrade your membership level? Please contact Customer Service at Service@asmusa.org.

Embargo Policy: A press release may be issued as soon as the manuscript is posted on the Spectrum Latest Articles webpage. The corresponding author will receive an email with the subject line "ASM Journals Author Services Notification" when the article is available online.

The ASM Journals program strives for constant improvement in our submission and publication process. Please tell us how we can improve your experience by taking this quick Author Survey.

Thank you for submitting your paper to Spectrum.

Sincerely,
Kenneth Gavina
Editor
Microbiology Spectrum
