# Supplementary material for: Rapid, low-cost, and portable molecular detection of HIV-1 subtype B and HCV 1a directly from blood
Source: Microbiol Spectr. 2026 Aug 5;14(9):e00416-26. doi: 10.1128/spectrum.00416-26 (PMC13532246; doi:10.1128/spectrum.00416-26)
Supplement: Supplementary file 2 [file spectrum.00416-26-s0001.docx]

**Supplementary Table 1. RT-LAMP primer sequences**

| **Target Pathogen** | **Primer**  **Name** | **Sequence (5’ 🡪 3’)** | **Source** | **Name in This Study** |
| --- | --- | --- | --- | --- |
| HCV | F3 | TCCCGGGAGAGCCATAGTG | Kargar et al., 2012 |  |
|  | B3 | CACTCGCAAGCACCCTATCAG |  |  |
|  | FIP | GATCCAAGAAAGGACCCGGTTTTTCTGCGGAACCGGTGAGTAC |  | HCV-K12 |
|  | BIP | CCTGGAGATTTGGGCGTGCTTTTAGTACCACAAGGCCTTTCGC |  |  |
|  | LF | TCATCCTGGCAATTCCG |  |  |
|  | LB | GCGAGACTGCTAGCCGAG |  |  |
| HCV | F3 | TCCCGGGAGAGCCATAGTG | This study, modified from Kargar et al., 2012 | HCV-K12M |
|  | B3 | CACTCGCAAGCACCCTATCAG |  |  |
|  | FIP | [BIO] GATCCAAGAAAGGACCCGGTTTTTCTGCGGAACCGGTGAGTAC |  |  |
|  | BIP | CCTGGAGATTTGGGCGTGCTTTTAGTACCACAAGGCCTTTCGC |  |  |
|  | LF | TCATCCTGGCAATTCCG |  |  |
|  | LB | [FAM] GCGAGACTGCTAGCCGAG |  |  |
| HCV | DN2-F3 | TCGAACAGCCTCCAGGACCC | Nyan & Swinson, 2016 |  |
|  | DN2-B3 | CACGGTCTACGAGACCTCC |  |  |
|  | DN2-FIP | CGGGCATTGAGCGGGTTTATCCTTTTGGTGAGTACACCGGAATTGC |  | HCV-NS16 |
|  | DN2-BIP | CGCGAGACTGCTAGCCGAGTTTTTAGCACCCTATCAGGCAGTAC |  |  |
|  | DN2-LF | AAAGGACCCGGTCATCCC |  |  |
|  | DN2-LB | GTCGCGAAAGGCCTTGTG |  |  |
| HCV | F3 | ACTCCACCATGAATCACTC | Witkowska McConnell et al., 2021 | HCV-WM21 |
|  | B3 | ATCAGGCAGTACCACAAGG |  |  |
|  | FIP | AGGCTGYACGACACTCATACCTGTGAGGAACTACTGTCTT |  |  |
|  | BIP | GGATMAACCCRCTCAATGCCTCGCRACCCAACRCTAC |  |  |
|  | LF | GCCATGGCTAGACGCT |  |  |
|  | LB | CGTGCCCCCGCRAGA |  |  |
| HIV-1 | F3 | GCCCATATCACCTAGAACTT | Zeng et al., 2014 |  |
|  | B3 | CTGGATGTAATCTATCCCATTC |  |  |
|  | FIP | CTGATAATGCTGAAAACATGGGTATCATGGGTAAAAGTAGTAGAAGAGA |  | HIV-1-Z14 |
|  | BIP | TTAAACACCATGCTAAACACAGTGGGCAGCTTCTTCATTGATGG |  |  |
|  | LF | TACTTCTGGGCTAAAAGC |  |  |
|  | LB | TCAAGCAGCCATGCAAATGTT |  |  |
| HIV-1 | F3 | GCCCATATCACCTAGAACTT | This study, modified from Zeng et al., 2014 | HIV-1-Z14M |
|  | B3 | CTGGATGTAATCTATCCCATTC |  |  |
|  | FIP | CTGATAATGCTGAAAACATGGGTATCATGGGTAAAAGTAGTAGAAGAGA |  |  |
|  | BIP | TTAAACACCATGCTAAACACAGTGGGCAGCTTCTTCATTGATGG |  |  |
|  | LF | [BIO] TACTTCTGGGCTAAAAGC |  |  |
|  | LB | [FAM] TCAAGCAGCCATGCAAATGTT |  |  |
| HIV-1 | F3 | GGTAAGAGATCAGGCTGAACATC | Hosaka et al., 2009 |  |
|  | B3 | GCTGGTCCTTTCCAAAGTGG |  |  |
|  | FIP | CCCCAATCCCCCCTTTTCTTAGACAGCAGTACAAATGGCA |  | HIV-1-H9 |
|  | BIP | AGTGCAGGGGAAAGAATAGTAGACCTGCTGTCCCTGTAATAAACCC |  |  |
|  | LF | TTAAAATTGTGGATGAAT |  |  |
|  | LB | GCAACAGACATACAAACTAAAG |  |  |
| HIV-1 | ACeIN-F3_c | CCTATTTGGAAAGGACCAGC | Ocwieja et al., 2015 | HIV-1-O15 |
|  | ACeIN-B3a | TCTTTGAAAYATACATATGRTG |  |  |
|  | ACeIN-B3b | AACATACATATGRTGYTTTACTA |  |  |
|  | ACeIN-FIPe | CTTGGTACTACYTTTATGTCACTAAARCTACTCTGGAAAGGTG |  |  |
|  | ACeIN-FIPf | CTTGGCACTACYTTTATGTCACTAAARCTYCTCTGGAAAGGTG |  |  |
|  | ACeIN-BIP | GGAYTATGGAAAACAGATGGCAGCCATGTTCTAATCYTCATCCTG |  |  |
|  | ACeIN-LF | TCTTGTATTACTACTGCCCCTT |  |  |
|  | ACeIN-LB | GTGATGATTGTGTGGCARGTA |  |  |
| HIV-1 | F3 | GGTTTATTACAGGGACAGCA | Rudolph et al., 2015 | HIV-1-R15 |
|  | B3 | ATCCTGTCTACTTGCCAC |  |  |
|  | FIP | CTTGTATTACTACTGCCCCTTCACGATCCACTTTGGAAAGGACC |  |  |
|  | BIP | TGACATAAAAGTAGTGCCAAGAAGATTTTACAATCATCACCTGCCATC |  |  |
|  | LF | CTTTCCAGAGAAGCTTTGCT |  |  |
|  | LB | AGCAAAGATCATTAGGGATTAT |  |  |
| A primer modification is shown in brackets. 5’ Biotin (BIO) and 5’ 6-Carboxyfluorescein (FAM). A primer set containing both of those modifications generated amplicons that were detectable by Milenia HybriDetect Universal Lateral Flow dipstick (TwistDx, Cambridge, UK) | | | | |

**Supplementary Table 2: Utility of a custom HCV CRISPR–Cas12a fluorescence assay to confirm target amplicons generated by HCV RT-LAMP.** Cold stored reagents were used to assemble customized master mixes. To increase the frequency of non-target amplicon generation and thereby enable evaluation of the custom HCV guide RNA (bolded orange), a higher final concentration of magnesium sulfate was used. The lower limit of detection (LLOD) of HCV RT-LAMP is bolded in green when 0.5M betaine was included, and bolded in red when betaine was omitted. The addition of betaine gave two benefits: it delayed the onset of non-target amplicons and resulted in a lower LLOD. An input amount of an RT-LAMP condition was designated as the LLOD based on two criteria. First, the LLOD is the lowest input amount, where all replicates have a time-to-positive (TTP) and a positive CRISPR–Cas12a detection. Second, all replicates of input amounts above the LLOD must also have a TTP and a positive CRISPR detection. The second criterion does not apply if the LLOD is the highest input amount within the input series.

| **RT-LAMP Condition** ^A^ | | | **Technical Replicates** | | | | |
| --- | --- | --- | --- | --- | --- | --- | --- |
| **Incubation Temp. (°C)** | **MgSO_4_ (mM)** | **Betaine (M)** | **Estimated Input IU** ^B^ | **Avg. TTP**  **(min)** | **TTP Range (min)** | **Detectable TTP** | **TTP and CRISPR Positive** ^C^ |
| 62 | 6 | **0** | **816** | 12 | 10-13 | 6/6 | 6/6 |
|  |  | 0.5 |  | 13 | 12-15 | 6/6 | 6/6 |
|  |  | 0 | 408 | 13 | 11-13 | **6/6** | **5/6** |
|  |  | 0.5 |  | 15 | 14-16 | 6/6 | 6/6 |
|  |  | 0 | 204 | 13 | 11-15 | **6/6** | **5/6** |
|  |  | 0.5 |  | 15 | 13-17 | 6/6 | 6/6 |
|  |  | 0 | ***102*** | 20 | 12-42 | **6/6** | **3/6** |
|  |  | ***0.5*** |  | 16 | 14-20 | 6/6 | 6/6 |
|  |  | 0 | 51 | 17 | 9-28 | **6/6** | **2/6** |
|  |  | 0.5 |  | 24 | 16-58 | **6/6** | **5/6** |
|  |  | 0 | 26 | 18 | 15-20 | **3/3** | **0/3** |
|  |  | 0.5 |  | 17 | 14-19 | 3/3 | 3/3 |
|  |  | 0 | 13 | 22 | 18-27 | **3/3** | **0/3** |
|  |  | 0.5 |  | 30 | 14-59 | **3/3** | **2/3** |
|  |  | 0 | 0 | 16 | 8-33 | **24/24** | **0/24** |
|  |  | 0.5 |  | 42 | 21-57 | **22/24** | **0/22** |

Abbreviations: IU, international units; temp., temperature; avg., average; TTP, time-to-positive; mins., minutes

^A^ Cold-stored RT-LAMP reagents and the HCV-K12 primer set were used to prepare a master mix (lyophilized master mix was not used). Reactions were incubated at 62°C for 60 minutes. The master mix contained a double-stranded DNA-binding fluorescence dye, enabling real-time monitoring of amplification. Time-to-positive was define as the time at which the amplification curve crossed a predefined fluorescence threshold.

^B^ The reference sample used was Amplichek I, Level 3 (Bio-Rad Laboratories Inc.), a human plasma matrix containing intact inactivated Hepatitis B Virus (HBV), Hepatitis C Virus (HCV) and Human Immunodeficiency Virus Type 1 (HIV-1). The estimated input in international units was calculated based on the HCV viral load provided by the manufacturer, the volume of sample used for nucleic extraction and the assumption that no viral RNA was lost during the extraction and elution process

^C^ A 2 µL aliquot of post-RT-LAMP product was used for CRISPR–Cas12a based fluorescence detection with custom designed HCV guide RNA 1

**Supplementary Table 3. Detection time and sensitivity of HCV and HIV-1 RT-LAMP primer sets screened using an off-the-shelf lyophilized master mix and serially diluted extracted viral RNA**

An input amount was designated as the LLOD based on two criteria: 1) all replicates at that input must produce a measurable time-to-positive (TTP), and 2) all replicates at higher input levels must also yield a TTP. The second criterion does not apply when the LLOD corresponds to the highest input amount tested in the input series.

| **Pathogen** | **RT-LAMP** | | | | |
| --- | --- | --- | --- | --- | --- |
|  | **Primer Set** | | **Technical Replicates** | | |
|  | **Source** | **Name in This Study** | **LLOD** | **Detectable TTP** | **Mean TTP** ± **SD (Min.)** |
| HIV-1 Subtype B | Hosaka et al., 2009 | HIV-1-H9 | 1000 copies | 3/3 | 23 ± 2 |
|  | Zeng et al., 2014 | HIV-1-Z14 | 1000 copies | 3/3 | 16 ± 2 |
|  | Rudolph et al., 2015 | HIV-1-R15 | ND | ND | ND |
|  | Ocwieja et al., 2015 | HIV-1-O15 | 6000 copies | 3/3 | 25 ± 7 |
| HCV 1a | Kargar et al., 2012 | HCV-K12 | 100 IU | 3/3 | 24 ± 2 |
|  | Nyan & Swinson, 2016 | HCV-NS16 | 400 IU | 3/3 | 17 ± 2 |
|  | Witkowska McConnell et al., 2021 | HCV-MW21 | 1600 IU | 3/3 | 28 ± 5 |

Abbreviations: LLOD, lower limited of detection; IU, international units; TTP, time-to-positive; mins., minutes; SD, standard deviation; N/A, not applicable; ND, not determined within input series

**Supplementary Table 4: Utility of a custom HIV-1 CRISPR–Cas12a fluorescence assay to confirm target amplicons generated by HIV-1 RT-LAMP.** Cold stored reagents were used to assemble customized master mixes containing varying concentrations of magnesium sulfate. Increasing the final concentration of magnesium sulfate increased the frequency of non-specific amplicons, which enable evaluation of the specificity of custom HIV-1 guide RNA 1 (bolded orange). Values bolded in green indicate the lower limit of detection (LLOD) of each HIV-1 RT-LAMP condition. An input amount was designated as the LLOD based on two criteria: 1) all replicates at that input must produce a measurable time-to-positive (TTP) and a positive CRISPR fluorescence signal; and 2) all replicates at higher input levels must also yield a TTP and a positive CRISPR signal. The second criterion does not apply when the LLOD corresponds to the highest input amount tested in the input series.

| **RT-LAMP Condition**^A^ | | | **Technical Replicates** | | | | |
| --- | --- | --- | --- | --- | --- | --- | --- |
|  |  |  | **Estimated Input Copies**^B^ | **Mean TTP (Mins.)** | **TTP Range (Mins.)** |  | **TTP and CRISPR Positive**^C^ |
| **Incubation Temp. (°C)** | **Betaine (M)** | **MgSO_4_ (mM)** |  |  |  | **Detectable TTP** |  |
| 62 | 0.5 | 4.5 | 500 | 56 | 56 | 1/4 | 1/1 |
|  |  | 6 |  | 24 | 19-27 | 4/4 | 4/4 |
|  |  | 7 |  | 22 | 20-23 | 4/4 | 4/4 |
|  |  | 4.5 | **250** | 53 | 47-59 | ***2/4*** | ***1/2*** |
|  |  | **6** |  | 38 | 29-55 | 4/4 | 4/4 |
|  |  | **7** |  | 30 | 26-36 | 4/4 | 4/4 |
|  |  | 4.5 | 125 | NA | NA | 0/4 | NA |
|  |  | 6 |  | 36 | 23-51 | 3/4 | 3/3 |
|  |  | 7 |  | 48 | 46-53 | ***4/4*** | ***0/4*** |
|  |  | 4.5 | 0 | 0 | 0 | 0/8 | NA |
|  |  | 6 |  | 50 | 35-57 | ***7/8*** | ***0/7*** |
|  |  | 7 |  | 50 | 33-55 | ***8/8*** | ***0/8*** |

Abbreviations: temp., temperature; conc., concentration; avg., TTP, time-to-positive; NA, not applicable; mins., minutes;

^A^ Cold-stored RT-LAMP reagents and the HIV-1-Z14 primer set were used to prepare a master mix (lyophilized master mix was not used). Reactions were incubated at 62°C for 60 minutes. The master mix contained a double-stranded DNA-binding fluorescence dye, enabling real-time monitoring of amplification. Time-to-positive was defined as the time at which the amplification curve crossed a predefined fluorescence threshold

^B^ A plasma sample positive for HIV-1 subtype B was obtained from the Virology Quality Assurance Program at the Duke Human Vaccine Institute. The estimated input copies was calculated based on the viral load provided by the vendor, the volume of plasma used for nucleic extraction, the final elution volume, and the assumption that no viral RNA was not lost during the extraction and elution process

^C^ A 2 µL aliquot of post-RT-LAMP product was used for CRISPR–Cas12a based fluorescence detection with custom designed HIV-1 guide RNA 1

**Supplementary Table 5: Custom-Designed Cas12a Guide RNA Sequences**

| Target Pathogen | Guide RNA Name | | Target Gene and Genomic Coordinates^A^ | Guide RNA Sequence (5’ 🡪 3’) |
| --- | --- | --- | --- | --- |
| HCV | HCV gRNA 1 | | 5’ UTR, 198 - 217 | UAAUUUCUACUAAGUGUAGAUUUGGAUCAACCCGCUCAAUG |
|  | HCV gRNA 2 | | 5’ UTR, 230 - 249 | UAAUUUCUACUAAGUGUAGAUGGCGUGCCCCCGCGAGACUG |
| HIV-1 | HIV-1 gRNA 1 | | gag, 866 - 885 | UAAUUUCUACUAAGUGUAGAUAAUCUUGUGGGGUGGCUCCU |
|  | HIV-1 gRNA 2 | | pol, 4347 - 4366 | UAAUUUCUACUAAGUGUAGAUCCCUGCACUGUACCCCCCAA |
|  | HIV-1 gRNA 3 | pol, 4570 - 4589 | | UAAUUUCUACUAAGUGUAGAUCAUAAUCCCUAAUGAUCUUU |

## ^A^ Reference sequences were AF033819.3 (HIV-1 subtype B) and AF009606.1 (HCV 1a)

**Supplementary Table 6. Compatibility of custom LbaCas12a guide RNAs with published RT-LAMP primer sets**

A guide RNA was deemed compatible with a primer set when: 1) a positive CRISPR detection (bolded green P) occurred with 2 µL of post-RT-LAMP material that contained amplicons and 2) a negative CRISPR detection (black N) occurred with 2 µL of post-RT-LAMP material that did not contain amplicons. A guide RNA was deemed incompatible with a primer set when a positive CRISPR detection (bold red P) occurred with 2 µL of post-RT-LAMP material that did not contain amplicons. To deduce whether a primer (carried over from the 2 µL of post-RT-LAMP material) bound with the guide RNA and Lba Cas12a complex and falsely activated the complex, sequence alignment analysis was performed. The RNA spacer sequence (20 nts) of the HIV-1 guide RNA 1 had 17 base pairs with the DN2-FIP primer (46 nts) from Nyan and Swinson, 2016. Meanwhile the RNA spacer sequence (20 nts) of the HCV guide RNA 3 had 19 base pairs with the loop B primer (22 nts) from Rudolph et al., 2015. Overall, the custom-designed guide RNAs were compatible with one or more published primer sets. It is likely that the custom-designed guide RNAs will be compatible with future RT-LAMP primer sets.

| **RT-LAMP Reaction^A^** | | | | **CRISPR-Lba Cas12a Fluorescence Detection Using a Custom-Designed Guide RNA^D^** | | | | |
| --- | --- | --- | --- | --- | --- | --- | --- | --- |
| **Primer Set** | | **Estimated Input^C^** | **TTP (mins.)** | **HCV Guide RNA 1** | **HCV Guide RNA 2** | **HIV-1 Guide RNA 1** | **HIV-1 Guide RNA 2** | **HIV-1 Guide RNA 3** |
| **Source** | **Target Gene and Genomic Coordinates^B^** |  |  |  |  |  |  |  |
| Kargar et al., 2012 | HCV 5'-UTR, 127-313 | 1600 IU | 16 | **P** | **P** | N | N | N |
|  |  | NF water | ND | N | N | N | N | N |
| DN2 from Nyan and Swinson., 2016 | HCV 5'-UTR, 103-337 | 1600 IU | 14 | **P** | **P** | N | N | N |
|  |  | NF water | ND | **P** | N | N | N | N |
| Witkowska McConnell et al., 2021 | HCV 5'-UTR, 24-297 | 1600 IU | 28 | **P** | **P** | N | N | N |
|  |  | NF water | ND | N | N | N | N | N |
| Zeng et al., 2014 | HIV-1 gag, 770-937 | 6000 copies | 16 | N | N | **P** | N | N |
|  |  | NF water | ND | N | N | N | N | N |
| Hosaka et al., 2009 | HIV-1 pol, 4267-4409 | 6000 copies | 18 | N | N | N | **P** | N |
|  |  | NF water | ND | N | N | N | N | N |
| Ocwieja et al., 2015 | HIV-1 pol, 4472-4626 | 6000 copies | 27 | N | N | N | N | **P** |
|  |  | NF water | ND | N | N | N | N | N |
| Rudolph et al., 2015 | HIV-1 pol, 4447-4615 | 6000 copies | 20 | N | N | N | N | **P** |
|  |  | NF water | ND | N | N | N | N | **P** |

**Abbreviations:** TTP, time to positive; mins., minutes; NF water, nuclease-free water; ND, no time to positive detected; P, positive CRISPR fluorescence detection; N, negative CRISPR fluorescence detection

^A^ Off-the-shelf lyophilized master mix with the addition of a primer set and betaine at a final concentration of 0.5 M. Reactions were incubated at 62°C for 60 minutes. The master mix contained a double-stranded DNA-binding fluorescence dye, enabling real-time monitoring of amplification. Time-to-positive was define as the time at which the amplification curve crossed a predefined fluorescence threshold. The primer set used from Ocwieja et al., 2015 was ACeIN-F3_c, ACeIN-B3a, ACeIN-B3b, ACeIN-FIPe, ACeIN-FIPf, ACeIN-BIP, ACeIN-LF, ACeIN-LB

^B^ Reference sequences were AF033819.3 (HIV-1 subtype B) and AF009606.1 (HCV 1a)

^C^ HIV-1 subtype B positive plasma (Virology Quality Assurance Program at the Duke Human Vaccine Institute) and HCV 1a positive plasma (SeraCare). The estimated input international units was calculated based on the reported viral load, the volume of plasma used for nucleic extraction and the assumption that no viral RNA was not lost during extraction and elution process

^D^ A 2 µL aliquot of post-RT-LAMP product was used for CRISPR–Cas12a based fluorescence detection with a custom designed guide RNA

**Supplementary Table 7. Lower limit of detection of HCV genotype 1a RNA under different HCV RT-LAMP conditions using an off-the-shelf lyophilized master mix**

Values bolded in green indicate the lower limit of detection (LLOD) of each incubation temperature and betaine concentration permutation. An input amount was designated as the LLOD based on two criteria: 1) all replicates at that input must produce a measurable time-to-positive (TTP) and a positive CRISPR fluorescence signal; and 2) all replicates at higher input levels must also yield a TTP and a positive CRISPR signal. The second criterion does not apply when the LLOD corresponds to the highest input amount tested in the input series.

| **Estimated input IU**^A^ | **RT-LAMP Condition**^B^ | | **Technical Replicates** | | | |
| --- | --- | --- | --- | --- | --- | --- |
|  | **Incubation Temp. (°C)** | **Betaine Conc. (M)** |  |  |  |  |
|  |  |  | **Detectable TTP** | **TTP and Negative CRISPR Detection**^C^ | **Mean TTP (Mins.)** | **TTP Range (Mins.)** |
| 1600 | 61 | 0 | 3/3 | none | 23 | 22-24 |
|  |  | 0.5 | 3/3 | none | 22 | 18-25 |
|  | 62 | 0 | 3/3 | none | 23 | 22-24 |
|  |  | 0.5 | 3/3 | none | 22 | 19-25 |
|  | 63 | 0 | 3/3 | none | 19 | 16-22 |
|  |  | 0.5 | 3/3 | none | 17 | 17-18 |
| 800 | 61 | 0 | 6/6 | none | 27 | 23-35 |
|  |  | 0.5 | 6/6 | none | 22 | 19-26 |
|  | **62** | **0** | **6/6** | none | **26** | **22-29** |
|  |  | 0.5 | 6/6 | none | 23 | 20-26 |
|  | **63** | 0 | 6/6 | none | 22 | 13-28 |
|  |  | **0.5** | **6/6** | none | **21** | **17-27** |
| 400 | **61** | **0** | **6/6** | none | **34** | **24-59** |
|  |  | **0.5** | **6/6** | none | **25** | **22-27** |
|  | 62 | 0 | 5/6 | none | 30 | 23-37 |
|  |  | 0.5 | 6/6 | none | 22 | 19-25 |
|  | **63** | **0** | **6/6** | none | **21** | **19-24** |
|  |  | 0.5 | 5/6 | none | 23 | 22-25 |
| 200 | 61 | 0 | 5/6 | none | 23 | 19-25 |
|  |  | 0.5 | 6/6 | 55 | 36 | 26-55 |
|  | **62** | 0 | 4/6 | none | 27 | 23-29 |
|  |  | **0.5** | **6/6** | none | **31** | **26-45** |
|  | 63 | 0 | 4/6 | none | 40 | 25-54 |
|  |  | 0.5 | 3/6 | none | 32 | 21-42 |
| 100 | 61 | 0 | 1/3 | none | 54 | 54 |
|  |  | 0.5 | 3/3 | none | 36 | 26-55 |
|  | 62 | 0 | 0/3 | none | NA | NA |
|  |  | 0.5 | 1/3 | none | 26 | 26 |
|  | 63 | 0 | 0/3 | none | NA | NA |
|  |  | 0.5 | 1/3 | none | 42 | 42 |
| 0 | 61 | 0 | 0/4 | none | NA | NA |
|  |  | 0.5 | 0/4 | none | NA | NA |
|  | 62 | 0 | 0/4 | none | NA | NA |
|  |  | 0.5 | 0/4 | none | NA | NA |
|  | 63 | 0 | 0/4 | none | NA | NA |
|  |  | 0.5 | 0/4 | none | NA | NA |

Abbreviations: temp., temperature; conc., concentration; TTP, time-to-positive; NA, not applicable; mins., minutes;

^A^ The reference sample was HCV 1a positive plasma (SeraCare, Milford, MA, USA). The estimated input international units was calculated based on the reported viral load, the volume of plasma used for nucleic extraction and the assumption that no viral RNA was not lost during extraction and elution process

^B^ Off-the-shelf lyophilized master mix with the HCV-K12 primer set. Reactions were incubated at the indicated temperature for 60 minutes. The master mix contained a double-stranded DNA-binding fluorescence dye, enabling real-time monitoring of amplification. Time-to-positive was define as the time at which the amplification curve crossed a predefined fluorescence threshold.

^C^ A 2 µL aliquot of post-RT-LAMP product was used for CRISPR–Cas12a based fluorescence detection with custom designed HCV guide RNA 1

**Supplementary Table 8. Lower limit of detection of HIV-1 subtype B RNA under different HIV-1 RT-LAMP conditions using an off-the-shelf lyophilized master mix**

Values bolded in green indicate the lower limit of detection (LLOD) of each incubation temperature and betaine concentration permutation. An input amount was designated as the LLOD based on two criteria: 1) all replicates at that input must produce a measurable time-to-positive (TTP) and a positive CRISPR fluorescence signal; and 2) all replicates at higher input levels must also yield a TTP and a positive CRISPR signal. The second criterion does not apply when the LLOD corresponds to the highest input amount tested in the input series

| **Estimated Input Copies**^A^ | **RT-LAMP Condition**^B^ | | **Technical Replicates** | | | |
| --- | --- | --- | --- | --- | --- | --- |
|  |  |  | **Detectable TTP** | **TTP and Negative CRISPR Detection**^C^ **(Mins.)** | **Mean TTP (Mins.)** | **TTP Range (Mins.)** |
|  | **Incubation Temp. (°C)** | **Betaine Conc. (M)** |  |  |  |  |
| 2000 | **61** | 0 | 3/3 | none | 19 | 16-20 |
|  |  | 0.5 | 3/3 | none | 22 | 19-24 |
|  | **62** | 0 | 3/3 | none | 23 | 18-26 |
|  |  | 0.5 | 3/3 | none | 22 | 18-26 |
|  | **63** | 0 | 3/3 | none | 24 | 18-31 |
|  |  | 0.5 | 3/3 | none | 24 | 21-25 |
| 1000 | **61** | 0 | 3/3 | none | 23 | 20-24 |
|  |  | 0.5 | 3/3 | none | 22 | 18-23 |
|  | **62** | 0 | 3/3 | none | 24 | 19-26 |
|  |  | 0.5 | 3/3 | none | 27 | 25-27 |
|  | ***63*** | **0** | **3/3** | **none** | **28** | **19-31** |
|  |  | 0.5 | 3/3 | none | 26 | 23-28 |
| 500 | **61** | 0 | 3/3 | none | 24 | 22-24 |
|  |  | 0.5 | 3/3 | none | 23 | 21-25 |
|  | **62** | 0 | 3/3 | none | 24 | 22-25 |
|  |  | 0.5 | 3/3 | none | 25 | 22-27 |
|  | **63** | 0 | 2/3 | none | 26 | 22-29 |
|  |  | 0.5 | 3/3 | none | 27 | 25-30 |
| 250 | ***61*** | **0** | **3/3** | **none** | **28** | **24-33** |
|  |  | **0.5** | **3/3** | **none** | **32** | **26-37** |
|  | ***62*** | **0** | **3/3** | **none** | **34** | **25-48** |
|  |  | **0.5** | **3/3** | **none** | **43** | **37-52** |
|  | ***63*** | 0 | 0/3 | none | NA | NA |
|  |  | **0.5** | **3/3** | none | **37** | **27-51** |
| 0 | **61** | 0 | 1/2 | 59 | 59 | 58 |
|  |  | 0.5 | 0/2 | none | NA | NA |
|  | **62** | 0 | 0/2 | none | NA | NA |
|  |  | 0.5 | 0/2 | none | NA | NA |
|  | **63** | 0 | 0/2 | none | NA | NA |
|  |  | 0.5 | 0/2 | none | NA | NA |

Abbreviations: temp., temperature; conc., concentration; TTP, time-to-positive; NA, not applicable; mins., minutes;

^A^ Plasma sample positive for HIV-1 subtype B from the Virology Quality Assurance Program at the Duke Human Vaccine Institute. The estimated input copies was calculated based on the viral load provided by the vendor, the volume of plasma used for nucleic extraction, the final elution volume, and the assumption that no viral RNA was not lost during the extraction and elution process.

^B^ Off-the-shelf lyophilized master mix with the HIV-1-Z14 primer set. Reactions were incubated at the indicated temperature for 60 minutes. The master mix contained a double-stranded DNA-binding fluorescence dye, enabling real-time monitoring of amplification. Time-to-positive was defined as the time at which the amplification curve crossed a predefined fluorescence threshold

^C^ A 2 µL aliquot of post-RT-LAMP product was used for CRISPR–Cas12a based fluorescence detection with custom designed HIV-1 guide RNA 1

### **Supplementary Table 9. Detection of Proviral HIV-1 DNA from 8E5 Genomic DNA Using Extraction-Free HIV-1 RT-LAMP and Lateral Flow Dipstick Readout**

| **Input 8E5 gDNA (ng)** | **RT-LAMP Technical Replicates** | | **Visible Test and Control Band on the Lateral Flow Dipstick** |
| --- | --- | --- | --- |
|  | **TTP ≤ 30 min** | **Mean TTP (min)** ± **SD** |  |
| 0 | 0/3 | N/A | No |
| 5.00 | 3/3 | 16.1 ± 0.4 | Yes |
| 2.50 | 3/3 | 17.0 ± 0.2 | Yes |
| 1.25 | 3/3 | 17.5 ± 0.2 | Yes |
| 0.63 | 3/3 | 18.4 ± 0.4 | Yes |
| 0.31 | 3/3 | 19.6 ± 0.9 | Yes |
| 0.16 | 3/3 | 21.4 ± 1.8 | Yes |
| 0.08 | 6/6 | 23.1 ± 3.8 | Yes |
| 0.04 | 2/3 | 34.9 ± 12.6 | NT |
| 0.02 | 1/3 | 31.9 ± 7.6 | NT |
| 0.01 | 0/3 | 33.4 ± 1.9 | NT |
| 0.005 | 1/3 | 42.8 ± 14.7 | NT |

Abbreviations: gDNA, genomic DNA; N/A, not applicable; TTP, time-to-positive; minutes, min; SD, standard deviation; NT, not tested by lateral flow dipstick

**Supplementary Table 10. Extraction-free HCV RT-LAMP performance across multiple HCV genotypes at a fixed estimated input of 1,000 IU**

| **Sample Type** | **Estimated Input (IU)** | **RT-LAMP Technical Replicates** | | |
| --- | --- | --- | --- | --- |
|  |  | **TTP Detected** | **Mean TTP ± SD (min)** | **TTP Detected and CRISPR-Positive** |
| Genotype 1a | 1000^A^ | 3/3 | 20.7 ± 2.6 | 3/3 |
| Genotype 1b |  | 3/3 | 18.3 ± 1.7 | 3/3 |
| Genotype 2b |  | 1/3 | 52 ± N/A | 0/3 |
| Genotype 3a |  | 3/3 | 47 ± 5 | 3/3 |
| Negative Plasma | 0 | 0/3 | N/A | 0/3 |

Abbreviations: N/A, not applicable; TTP, time-to-positive; minutes, min; SD, standard deviation

^A^ Even at high input levels of extracted 2b or 3a RNA (1000 IU, equivalent to 5.60 log_10_ IU/mL in a contrived blood sample), none of the triplicate reactions generated a detectable TTP within 30 min.

**Supplementary Table 11. Alignment of the HCV-K12 RT-LAMP primer set to reference sequences representing multiple HCV genotypes**
Alignment analysis showing the number of nucleotide mismatches between the HCV RT-LAMP primer set (F3, F2, F1c, B1c, B2, B3, LF, and LB) and representative reference sequences for selected HCV genotypes. Total mismatches represent the sum across all primers.

| **HCV genotype** | **Reference sequence (accession)** | **Total mismatches** | **F3** | **F2** | **LF** | **F1c** | **B1c** | **LB** | **B2** | **B3** |
| --- | --- | --- | --- | --- | --- | --- | --- | --- | --- | --- |
| HCV 1a | AF00606.1 | 3 | 0 | 0 | 1 | 1 | 0 | 1 | 0 | 0 |
| HCV 1a | EU255992.1 | 2 | 0 | 0 | 1 | 1 | 0 | 0 | 0 | 0 |
| HCV 1b | M58335.1 | 1 | 0 | 0 | 1 | 0 | 0 | 0 | 0 | 0 |
| HCV 2b | AF238486.1 | 11 | 0 | 0 | 4 | 2 | 4 | 1 | 0 | 0 |
| HCV 3a | MN231293.1 | 11 | 0 | 0 | 4 | 1 | 3 | 3 | 0 | 0 |

**Supplementary Table 12. Positive control gBlock sequences**

| **Target** | **Sequence (5’ 🡪 3’)** | **Accession Number** |
| --- | --- | --- |
| HCV genotype 1a  5’ UTR | GCCAGCCCCCTGATGGGGGCGACACTCCACCATGAATCACTCCCCTGTGAGGAACTACTGTCTTCACGCAGAAAGCGTCTAGCCATGGCGTTAGTATGAGTGTCGTGCAGCCTCCAGGACCCCCCCTCCCGGGAGAGCCATAGTGGTCTGCGGAACCGGTGAGTACACCGGAATTGCCAGGACGACCGGGTCCTTTCTTGGATAAACCCGCTCAATGCCTGGAGATTTGGGCGTGCCCCCGCAAGACTGCTAGCCGAGTAGTGTTGGGTCGCGAAAGGCCTTGTGGTACTGCCTGATAGGGTGCTTGCGAGTGCCCCGGGAGGTCTCGTAGACCGTGCACCATGAGCACGAATCCTAAACCTCAAAGAAAAACCAAACGTAACACCAACCGTCGCCCACAGGACGTCAAGTTCCCGGGTGGCGGTCAGATCGTTGGTGGAGTTTACTTGTTGCCGCGCAGGGGCCCTAGATTGGGTGTGCGCGCGACGAGGAAGACTTCCGAGCGGTCGCAACCTCGAGGTAGACGTCAGCCTATCCCCAAGGCACGTCGGCCCGAGGGCAGGACCTGGGCTCAGCCCGGGTACCCTTGGCCCCTCTATGGCAATGAGGGTTGCGGGTGGGCGGGATGGCTCCTGTCTCCCCGTGGCTCTCGGCCTAGCTGGGGCCCCACAGACCCCCGGCGTAGGTCGCGCAATTTGGGTAAGGTCATCGATACCCTTACGTGCGGCTTCGCCGACCTCATGGGGTACA | AF009606.1 |
| HIV-1  gag | AGGCTGTAGACAAATACTGGGACAGCTACAACCATCCCTTCAGACAGGATCAGAAGAACTTAGATCATTATATAATACAGTAGCAACCCTCTATTGTGTGCATCAAAGGATAGAGATAAAAGACACCAAGGAAGCTTTAGACAAGATAGAGGAAGAGCAAAACAAAAGTAAGAAAAAAGCACAGCAAGCAGCAGCTGACACAGGACACAGCAATCAGGTCAGCCAAAATTACCCTATAGTGCAGAACATCCAGGGGCAAATGGTACATCAGGCCATATCACCTAGAACTTTAAATGCATGGGTAAAAGTAGTAGAAGAGAAGGCTTTCAGCCCAGAAGTGATACCCATGTTTTCAGCATTATCAGAAGGAGCCACCCCACAAGATTTAAACACCATGCTAAACACAGTGGGGGGACATCAAGCAGCCATGCAAATGTTAAAAGAGACCATCAATGAGGAAGCTGCAGAATGGGATAGAGTGCATCCAGTGCATGCAGGGCCTATTGCACCAGGCCAGATGAGAGAACCAAGGGGAAGTGACATAGCAGGAACTACTAGTACCCTTCAGGAACAAATAGGATGGATGACAAATAATCCACCTATCCCAGTAGGAGAAATTTATAAAAGATGGATAATCCTGGGATTAAATAAAATAGTAAGAATGTATAGCCCTACCAGCATTCTGGACATAAGACAAGGACCAAAGGAACCCTTTAGAGACTATGTAGACCGGTTCTATAAAACTCTAAG | AF033819.3 |

**Supplementary Figure 1. Distribution of time-to-positive (TTP) values for non-target amplicons in no-template controls from HCV RT-LAMP reactions under variable betaine concentrations.** Individual TTP values and CRISPR detection results are shown for replicates described in Table S2. These reactions utilized a cold-stored master mix with elevated magnesium (6 mM) to deliberately promote non-specific amplification in no-template control reactions. For the 0 M betaine group (n=24), all replicates exhibited non-target amplification. For the 0.5 M betaine group, 20 of 22 technical replicates (n=22) exhibited a TTP within the 60-minute runtime; the two replicates that showed no amplification were not plotted as they lack a TTP value. Horizontal bars and adjacent numerical labels represent the mean TTP of the amplifying replicates within each group (15.7 min for 0 M betaine and 41.7 min for 0.5 M betaine). These data illustrate that the addition of 0.5 M betaine not only reduces the frequency of non-target amplicons but also significantly delays their time trajectory, providing greater separation between TTP values of target and non-target amplification.

**Supplementary Figure 2. Specificity of extraction-free RT-LAMP assays for HCV and HIV-1.** Contrived blood samples containing HCV genotype 1a or HIV-1 subtype B were tested in both RT-LAMP assays. Each assay exclusively detected its corresponding target, with no cross-amplification observed between HCV and HIV-1 reactions. Viral load of contrived blood samples was described in Figure 4.

**Supplementary Figure 3. Lower limit of detection of HCV genotypes 1b, 2b and 3a using optimized extraction-free HCV RT-LAMP with lateral flow readout.** Contrived whole-blood samples were prepared using patient-derived HCV genotype 1b, 2b, or 3a plasma reference material. Viral loads were quantified using the Xpert HCV VL Fingerstick assay. Samples were then analyzed using optimized extraction-free HCV RT-LAMP, and the presence of target amplicons was assessed after a 30 min incubation at 62 °C using CRISPR–Cas12a fluorescence detection and lateral flow readout, as described in Figure 1.
